# Supplementary material for: Highly sensitive switching of solid-state luminescence by controlling intersystem crossing
Source: Nat Commun. 2018 Aug 2;9:3044. doi: 10.1038/s41467-018-05476-y (PMC6072740; doi:10.1038/s41467-018-05476-y)
Supplement: Supplementary file 1 — Supplementary Information [file 41467_2018_5476_MOESM1_ESM.docx]

**Supplementary Information**

**Highly sensitive switching of solid-state luminescence by controlling intersystem crossing**

Zhao et al.

**Supplementary Methods**

Benzophenone and triphenylamine was purchased from Alfa Aesar. Tetrakis(triphenylphosphine) palladium (Pd(PPh_3_)_4_), potassium carbonate (K_2_CO_3_), titanium tetrachloride (TiCl_4_), zinc dust (Zn), ammonium chloride (NH_4_Cl), nitric acid (HNO_3_) (68%), fuming nitric acid (98%), glacial acetic acid (AcOH), dichloromethane (DCM), chloroform (CHL), hexane, 1,2-dichloroethane, sodium bicarbonate and anhydrous magnesium sulfate were purchased from Sinopharm Chemical Reagent Co., Ltd. Tetrahydrofuran (THF) was purchased from Aldrich and distilled under normal pressure from sodium benzophenone ketyl under argon immediately prior to use.

The self-McMurry coupling reaction was carried out under a nitrogen atmosphere using Schlenk technique. ^1^H-NMR and ^13^C-NMR spectra were recorded using a Bruker AV 400 Spectrometer at 400 and 100 MHz in DMSO-d_6_ or CDCl_3_ solutions, respectively. Tetramethylsilane was used as the internal standard. A Bruker microTOF-Q Ⅱ mass spectrometer was used to obtain exact molecular mass. Fluorescence spectra of all samples were measured by a Horiba Fluoromax 4 spectrofluorometer. Fluorescence quantum yields of solids were recorded on Horiba Fluoromax 4 at room temperature with a calibrated integrating sphere system. Fluorescence lifetimes were measured with Hamamatsu Quantaurus-Tau C11367-11 at room temperature. UV-vis absorption spectra were recorded on a TU-1901. Differential scanning calorimetry (DSC) was carried out using a Mettler DSC 1 instrument at a scaning rate of 10 Kmin^-1^. Thermal gravity analysis (TGA) was carried out using a METTLER STARESW 9.30 at a scaning rate of 10 Kmin^-1^ in the atmosphere of nitrogen. Powder X-Ray diffraction (PXRD) patterns were performed on a X’Pert PRO MPD diffractometer with Cu Kα radiation (λ = 1.5418 Å) at 25 ^o^C (scan range: 4.5-50^o^). Single crystal data was collected on a Bruker Smart APEXII CCD diffractometer using graphite monochromated Mo Kα radiation (λ=0.71070 Å). Melting points were measured by DSC analysis. The thermal annealing processes were carried out in oven or heated by a handed heat gun. The microscopy images were taken by fluorescence microscope ZEISS Imager M1 under UV irradiation at 400 nm. All normal photographs were recorded on a FinePix S7000 digital camera. Refractive indices were determined on a JA Woolam Variable Angle Ellipsometry System with a wavelength tunable from 400 to 900 nm. Polarizing microscope imaging was captured by an OLYMPUS BX51 (Olympus Optical Co Ltd, Tokyo, Japan) polarizing microscope coupled with a digital camera (Olympus, DP70).

Mean decay times (τ) were obtained from individual lifetimes *τ*_i_ and amplitudes *a*_i_ of multi-exponential evaluation by Supplementary Equation (1):


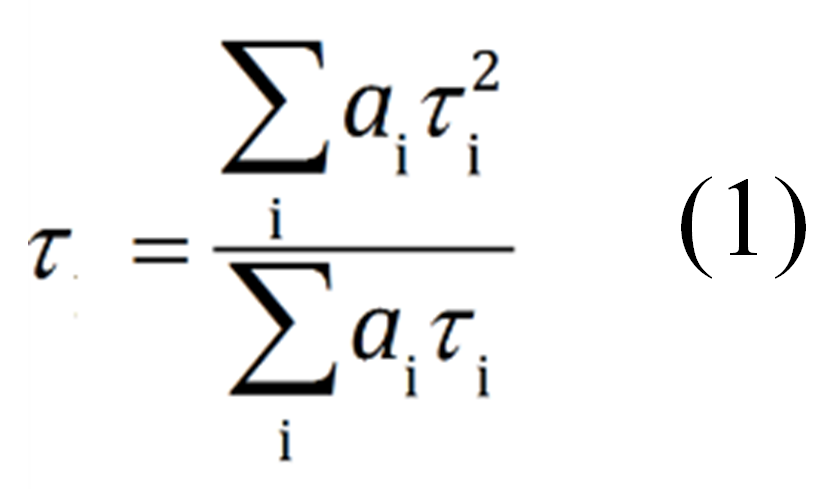


**Synthesis**

**Supplementary Figure 1.** Synthetic routes for TPA-1N, TPA-2N, TPE-1N, TPE-2N, TPE-3N and TPE-4N.

4-nitro-N,N-diphenylaniline (TPA-1N)

To a mixture of nitric acid (68%, 4 ml) and glacial acetic acid (10 ml) was dropwise added a solution of triphenylamine (1.23 g, 5 mmol, 100 ml DCM) at 0 °C and the reaction mixture was stirred at room temperature for 3 h. The mixture was poured into 100 mL of water, and extracted with 50 ml DCM. The combined organic layers were washed with sodium bicarbonate solution and desiccated with anhydrous magnesium sulfate for two hours, and then filtered. The solvent were removed by evaporation. Finally, the residue was recrystallized from DCM/hexane to give the product as yellow crystals (1.16 g, 85%). Melting point: 145.8 °C. ^1^H-NMR (400 MHz, DMSO-d_6_): *δ*: 8.07 (d, J = 9.3 Hz, ArH, 2H), 7.45 (t, J = 7.8 Hz, ArH, 4H), 7.31-7.25 (m, ArH, 6H), 6.80 (d, J = 9.6 Hz, ArH, 2H). ^13^C-NMR (100 MHz, DMSO-d_6_): *δ*: 116.62, 125.49, 126.07, 126.68, 130.05, 138.77, 144.76, 153.11. HRMS (ESI, *m*/*z*): calcd. for C_18_H_14_N_2_O_2_, 290.3220. Found, 290.3218. Elemental analysis (calcd., found for C_18_H_14_N_2_O_2_): C (74.47, 74.45), H (4.86, 4.89), N (9.65, 9.70).

4-nitro-N-(4-nitrophenyl)-N-phenylaniline (TPA-2N)

To a mixture of nitric acid (68%, 5 ml) and glacial acetic acid (10 ml) was dropwise added a solution of triphenylamine (1.23 g, 5 mmol, 100 ml DCM) at 0 °C and the reaction mixture was stirred at room temperature for 1 h. Another portion of nitric acid (68%, 5 ml) was added to the reaction solution and the reaction stirred for 2 h. The mixture was poured into 100 mL of water, and extracted with 50 ml DCM. The combined organic layers were washed with sodium bicarbonate solution and desiccated with anhydrous magnesium sulfate for two hours, and then filtered. The solvent were removed by evaporation. Finally, the residue was recrystallized from DCM/hexane to give the product as yellow crystals (1.51 g, 90%). Melting point: 198.2 °C. ^1^H-NMR (400 MHz, DMSO-d_6_): *δ*: 8.19 (d, J = 9.3 Hz, ArH, 4H), 7.51 (t, J = 7.5 Hz, ArH, 2H), 7.37 (t, J = 6.9 Hz, ArH, 2H), 7.27 (d, J = 7.5 Hz, ArH, 2H), 7.20 (d, J = 9.0 Hz, ArH, 4H). ^13^C-NMR (100MHz, DMSO-d_6_): *δ*: 122.25, 125.41, 126.99, 127.27, 130.45, 141.83, 144.27, 151.44. HRMS (ESI, *m*/*z*): calcd. for C_26_H_18_N_2_O_4_, 335.3190. Found, 335.3181. Elemental analysis (calcd., found for C_18_H_13_N_3_O_4_): C (64.48, 64.51), H (3.91, 3.96), N (12.53, 12.48).

Tetraphenylethene (TPE)

As a typical self-McMurry coupling reaction, a 500 mL two necked flask equipped with a magnetic stirrer was charged with zinc dust (13.2 g, 200 mmol) and 200 mL THF under nitrogen atmosphere. The mixture was cooled to 0 °C, and TiCl_4_ (11 mL, 100 mmol) was added slowly by a syringe. The mixture was refluxed for 2.5 h and cooled to 0 °C. Benzophenone (14.6 g，80 mmol) in THF (100 mL) was added to the mixture. Then the mixture was refluxed until TLC showing complete conversion. The reaction was quenched with saturated aqueous NH_4_Cl solution, and extracted with DCM. The organic layer was desiccated with anhydrous magnesium sulfate for two hours, and then filtered. The solvent were removed by evaporation. Finally, the resulting residue was purified by recrystallization from acetone to give the products as white crystals (12.7 g, 96%). ^1^H NMR (400MHz, CDCl_3_): *δ*: 7.05-7.08 (m, Ar-8H), 7.10-7.13 (m, Ar-12H). ^13^C NMR (100 MHz, CDCl_3_): *δ*: 143.82, 144.06, 131.43, 127.75, 126.51.

(2-(4-nitrophenyl)ethene-1,1,2-triyl)tribenzene (TPE-1N)

To a mixture of nitric acid (68%, 12 ml) and glacial acetic acid (180 ml) was dropwise added a solution of tetraphenylethene (3.32 g, 10 mmol, 100 ml DCM) at 0 °C and the reaction mixture was stirred at room temperature for 15 min. Another portion of nitric acid (68%, 12 ml) was added to the reaction solution and the reaction stirred for 15 min. The mixture was poured into 100 mL of water, and extracted with 150 ml DCM. The combined organic layers were washed with sodium bicarbonate solution and desiccated with anhydrous magnesium sulfate for two hours, and then filtered. The solvent were removed by evaporation. Finally, the residue was recrystallized from DCM/hexane to give the product as yellow solid (3.8 g, 95%). Melting point: 76.3 °C. ^1^H NMR (400 MHz, DMSO-d_6_): *δ*: 7.97-7.99 (d, J = 8.68 Hz, Ar-2H) 7.11-7.22 (m, Ar-11H), 6.98-6.99 (m, Ar-6H). ^13^C-NMR (100 MHz, DMSO-d_6_): *δ*: 122.97, 126.97, 127.17, 127.55, 127.53, 127.81, 127.95, 128.05, 128.18, 130.49, 130.62, 131.85, 138.67, 142.07, 142.23, 142.36, 143.09, 145.56, 150.5. HRMS (ESI, *m*/*z*): calcd. for C_26_H_19_NO_2_, [M + Na]^+^ 400.1380. Found, 400.1308. Elemental analysis (calcd., found for C_26_H_19_NO_2_): C (82.58, 82.74), H (4.69, 5.07), N (3.68, 3.71).

4,4'-(2,2-diphenylethene-1,1-diyl)bis(nitrobenzene) (TPE-2N)

To a mixture of nitric acid (68%, 12 ml) and glacial acetic acid (180 ml) was dropwise added a solution of tetraphenylethene (3.32 g, 10 mmol, 100 ml DCM) at 0 °C and the reaction mixture was stirred at room temperature for 30 min. A portion of nitric acid (68%, 12 ml) was added to the reaction solution and the reaction stirred for 30 min. Another portion of nitric acid (68%, 12 ml) was added to the reaction solution and the reaction stirred for 1.5 h. The mixture was poured into 100 mL of water, and extracted with 150 ml DCM. The combined organic layers were washed with sodium bicarbonate solution and desiccated with anhydrous magnesium sulfate for two hours, and then filtered. The solvent were removed by evaporation. Finally, the residue was recrystallized from DCM/hexane to give the product as yellow crystals (4.22 g, 95%). Melting point: 200.6 °C. ^1^H-NMR (400MHz, DMSO-d_6_): *δ*: 8.03-8.05 (d, J = 8.76 Hz, Ar-4H), 7.21-7.29 (m, Ar-4H), 7.19-7.20 (m, Ar-6H), 7.03-7.05 (m, Ar-4H). ^13^C-NMR (100 MHz, DMSO-d_6_): *δ*: 123.22, 127.64, 128.12, 130.55, 132.01, 136.78, 141.58, 145.42, 145.90, 149.29. HRMS (ESI, *m*/*z*): calcd. for C_26_H_18_N_2_O_4_, [M + Na]^+^ 445.1233. Found, 445.1159. Elemental analysis (calcd., found for C_26_H_18_N_2_O_4_): C (73.39, 73.92), H (4.20, 4.29), N (6.47, 6.63).

4,4',4''-(2-phenylethene-1,1,2-triyl)tris(nitrobenzene) (TPE-3N)

To a mixture of nitric acid (68%, 48 ml) and glacial acetic acid (60 ml) was dropwise added a solution of tetraphenylethene (3.32 g, 10 mmol, 100 ml DCM) at 0 °C and the reaction mixture was stirred at room temperature for 4 h. The mixture was poured into 100 mL of water, and extracted with 150 ml DCM. The combined organic layers were washed with sodium bicarbonate solution and desiccated with anhydrous magnesium sulfate for two hours, and then filtered. The solvent were removed by evaporation. Finally, the residue was recrystallized from CHL/hexane to give the product as yellow needlelike crystal (4.16 g, 85%). Melting point: 97.9 °C. ^1^H-NMR (400 MHz, DMSO-d_6_): *δ*: 8.06-8.10 (m, Ar-6H), 7.30-7.33 (m, Ar-6H), 7.22-7.24 (m, Ar-3H), 7.05-7.07 (m, Ar-2H). ^13^C-NMR (100 MHz, DMSO-d_6_): *δ*: 123.26, 123.34, 123.48, 128.02, 128.37, 130.55, 131.87, 131.95, 132.03, 138.90, 140.66, 143.12, 146.19,146.33,148.17,148.53,148.57. HRMS (ESI, *m*/*z*): calcd for C_26_H_17_N_3_O_6_, [M + Na]^+^ 490.1118. Found, 490.1135. Elemental analysis (calcd., found for C_26_H_17_N_3_NaO_6_): C (60.74, 60.81), H (3.44, 3.67), N (8.85, 8.99).

1,1,2,2-tetrakis(4-nitrophenyl)ethane (TPE-4N)

To a mixture of fuming nitric acid (98%, 100 ml) and glacial acetic acid (100 ml) was added tetraphenylethylene (9.94 g, 30 mmol) in portions at 0 °C and the reaction mixture was stirred at room temperature for 2 h. The mixture was poured into 100 mL of water, and extracted with 150 ml DCM. The combined organic layers were washed with sodium bicarbonate solution and desiccated with anhydrous magnesium sulfate for two hours, and then filtered. The solvent were removed by evaporation. Finally, the residue was recrystallized from dioxane to give the product as yellow crystals (12.3 g, 80%). Melting point: 301.6 °C. ^1^H-NMR (400 MHz, DMSO-d_6_): *δ*: 8.06-8.08 (d, J = 8.08 Hz, Ar-8H), 7.19-7.21 (d, J = 8.08 Hz, Ar-8H). ^13^C-NMR (100 MHz, DMSO-d_6_): *δ*: 123.92, 131.80, 141.55, 147.13, 147.34. HRMS (ESI, *m*/*z*): calcd. for C_26_H_16_N_4_O_8_, [M + H]^+^ 513.1; Found, 513.1. Elemental analysis (calcd., found for C_26_H_16_N_4_O_8_ ): C (60.73, 60.94), H (3.14, 3.15), N (10.63, 10.93).

Phenyl(4'-(1,2,2-triphenylvinyl)-[1,1'-biphenyl]-4-yl)methanone (TPE-BP)

Pd(PPh_3_)_4_ (0.11 g, 0.01 mmol) was added into a stirred mixture of (4-(1,2,2-triphenylvinyl)phenyl) boronic acid (TPE-BA)^1^ (0.38 g, 1 mmol), 4-bromobenzophenone (0.26 g, 1 mmol) and K_2_CO_3_ (0.69 g, 5 mmol) in 50 mL of THF and 8 mL of water under nitrogen. The mixture was heated to 90 °C for 12 h. After cooling to room temperature, the solution was extracted with DCM (100 mL) twicely, washed with water and dried over Na_2_SO_4_. After filtration and solvent evaporation under reduced pressure, the product was purified by silica-gel column chromatography using hexane/dichloromethane as the eluent. TPE-BP was obtained in a 95% yield (0.49 g) as a white powder after recrystallization from a mixture of DCM/hexane. ^1^H-NMR (400 MHz, CDCl_3_): *δ*: 8.86-7.80 (m, 4H), 7.67–7.58 (m, 3H), 7.51–7.48 (m, 2H), 7.42–7.40 (m, 2H), 7.15–7.03 (m, 17H). ^13^C-NMR (100 MHz, CDCl_3_): *δ* 196.4, 144.7, 143.9, 143.6, 143.5, 141.5, 140.3, 137.8, 137.5, 136.0, 132.4, 132.0, 131.4, 131.3, 130.7, 130.0, 128.3, 127.8, 127.6, 126.7, 126.6, 126.5, 126.4. HRMS (ESI, *m*/*z*): calcd. for C_29_H_28_O, 512.6520. Found, 512.6527. Elemental analysis (calcd., found for C_29_H_28_O): C (91.37, 91.30), H (5.51, 5.57).

Phenyl(4'-(1,2,2-triphenylvinyl)-[1,1'-biphenyl]-4-yl)methanone (2TPE-BP)

Pd(PPh_3_)_4_ (0.11 g, 0.01 mmol) was added into a stirred mixture of TPE-BA (0.84 g, 2.2 mmol), 4,4'-dibromobenzophenone (0.34 g, 1 mmol) and K_2_CO_3_ (1.4 g, 10 mmol) in 50 mL of THF and 8 mL of water under nitrogen. The mixture was heated to 90 °C for 12 h. After cooling to room temperature, the solution was extracted with DCM (100 mL) twicely, washed with water and dried over Na_2_SO_4_. After filtration and solvent evaporation under reduced pressure, the product was purified by silica-gel column chromatography using DCM/hexane as the eluent. 2TPE-BP was obtained in a 90% yield (0.76 g) as a white powder after recrystallization from a mixture of DCM/hexane.^1^H-NMR (400 MHz, CDCl_3_): *δ*: 7.87-7.85 (d, J = 8.4 Hz, 4H), 7.67–7.65 (d, J = 8.4 Hz, 4H), 7.42–7.40 (d, J = 8.4 Hz, 4H), 7.14–7.03 (m, 34H). ^13^C-NMR (100 MHz, CDCl_3_): *δ* 195.9, 144.6, 143.9, 143.6, 143.5, 140.3, 137.6, 136.2, 132.0, 131.4, 131.3(2), 130.6, 127.8, 127.7, 126.6, 126.7, 126.6, 126.4. HRMS (ESI, *m*/*z*): calcd. for C_65_H_40_O, 843.0820. Found, 843.0825. Elemental analysis (calcd., found for C_65_H_40_O): C (92.60, 92.64), H (5.50, 5.54).





**Supplementary Figure 2**. PL spectra of luminogens of (a) TPA-1N, (b) TPA-2N, (c) TPE-1N, (d) TPE-2N, (e) TPE-3N and (f) TPE-4N in acetonitrile/water mixtures with varied water fractions (*f*_w_).

**
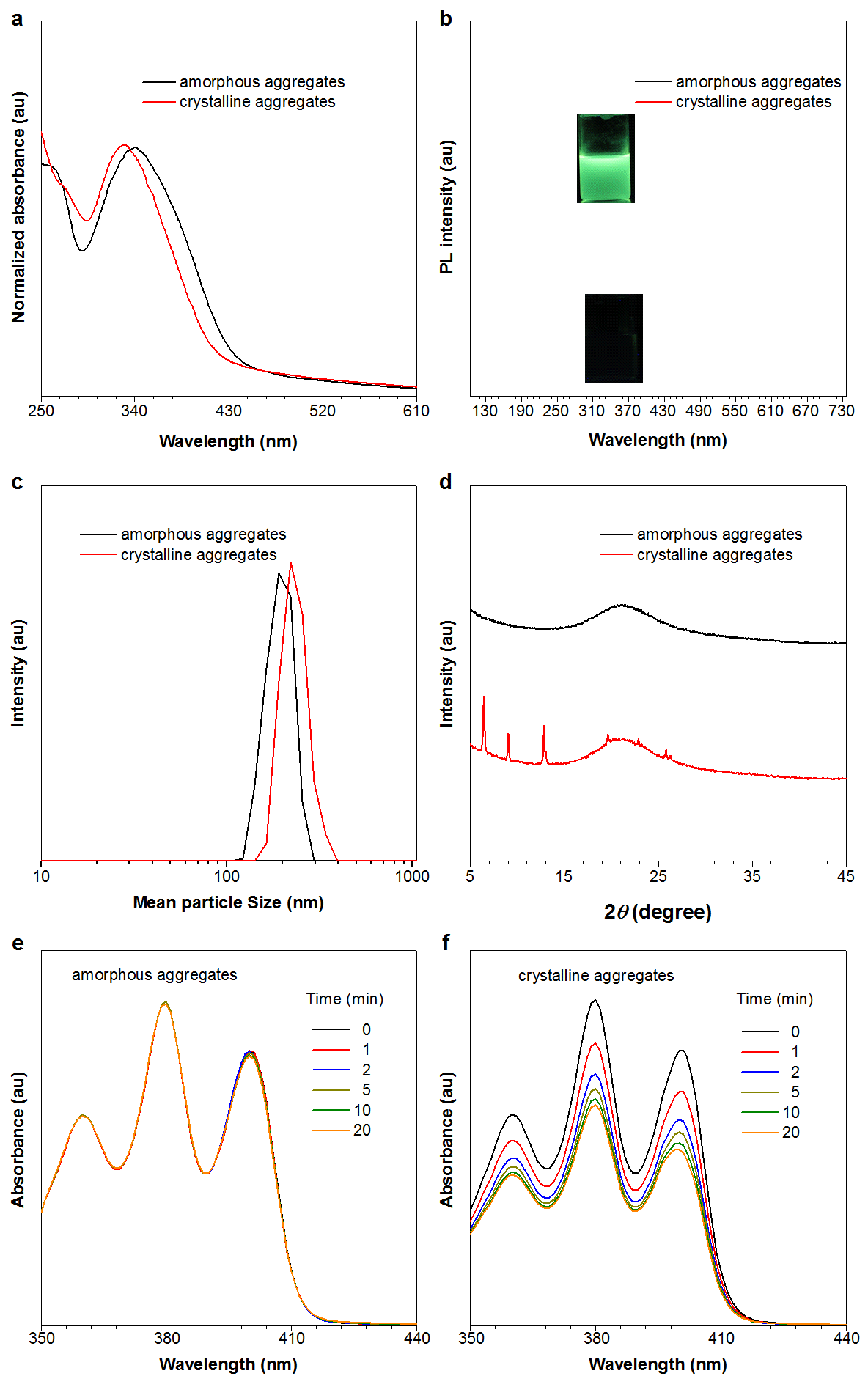
**

**Supplementary Figure 3.** (a) UV–Vis absorption and (b) PL spectra of TPE-4N in crystalline and amorphous states in water upon excitation at 365 nm; the insets of (b) show the photographs of the samples under UV excitation. (c) hydrodynamic mean particle size distribution of TPE-FN nanoaggregates (amorphous) and nanocrystals (crystalline) by dynamic light scattering; (d) PXRD patterns of amorphous and crystalline nanoaggregates. UV-vis spectra of ABDA in the presence of (e) amorphous and (f) crystalline nanoaggregates of TPE-4N under white light irradiation in water. [TPE-4N] = 0.4×10^−3^ M, [ABDA] = 5×10^−5^ M, time interval for recording the UV-vis spectra: 60 s.

Amorphous nanoaggregate fabrication^10^.

Amorphous nanoaggregates were prepared by adding 2 mL of TPE-4N (2 × 10^−3^ M in THF) to 8 mL of water under bath ultrasonication for 30 s. THF was then removed by simple evaporation under fume hood during 1 day.

Nanocrystalline aggregates fabrication^10^.

Crystalline nanoaggregates were prepared by adding 2 mL of TPE-4N (2 × 10^−3^ M in acetone) to 8.0 mL of water under bath ultrasonication for 3 min. After 2 h, crystallization was completed. Acetone was then removed by simple evaporation under fume hood during 1 day.

Detection of singlet oxygen generated by different TPE-4N particles.

9,10-anthracenediyl-bis(methylene)dimalonic acid (ABDA) is a reagent used to detect singlet oxygen. The absorbance intensity of ABDA decreased fast after crystalline nanoaggregate was added into the solution, while the absorbance intensity shown no evident change after amorphous nanoaggregate was added into the solution. Those results suggest that crystalline TPE-4N is a singlet oxygen promoter which can easily go to triplet state, while amorphous TPE-4N is not a singlet oxygen promoter which cannot easily go to the triplet state.





**Supplementary Figure 4.** (Up) Photos of emission switching on and off of TPA-1N powders through grinding and fuming. (Down) (a) PL spectra and (b) PXRD of TPA-1N through repeating grinding and solvent fuming in five repeating cycles.





**Supplementary Figure 5**. (Up) Photos of emission switching on and off of TPA-2N powders. (Down) (a) PL spectra and (b) PXRD of TPA-2N through repeating grinding and solvent fuming in five repeating cycles.





**Supplementary Figure 6**. (a-f) Photos and (g, h) PL spectra of luminongen TPE-1N (a, b, c, g) and TPE-2N (d, e, f, h): (a, d) crystals, (b, e) amorphous solids, and (c, f) ground powders.





**Supplementary Figure 7.** (Up) Photos of emission switching on and off of TPE-4N powders. (a) PL spectra of TPE-4N through repeating grinding and acetone fuming in five repeating cycles. (b) PL spectra of TPE-4N through repeating grinding and heating in five repeating cycles.





**Supplementary Figure 8.** (a-c) Photos of luminogen TPE-3N under room light (Up) and UV light (Down) (a) single crystals grown from chloroform solution, (b) amorphous solids and (c) ground powders. (d) PL spectra, (e) PXRD patterns of samples a-c. (f) DSC of amorphous, ground solid and crystals of TPE-3N. (g) ^1^H-NMR of TPE-3N crystals dissolved in DMSO-*d*_6_. The ^1^H-NMR spectra of the crystals obtained through recrystallization from solution indicate the existence of solvent molecule chloroform (CHL).





**Supplementary Figure 9**. (a-i) Photos of luminogen TPE-4N under room light (Up) and UV light (Down): (a) single crystals grown from acetone, single crystals heated at (b) 115 °C for 1 hour and then (c) 150 °C for 1 min, (d) solids obtained through quenching melts of TPE-4N, (e) ground powders, (f) ground powders after heated at 160 °C for 3 min, (g) ground powders after fumed with acetone for 3 min, sample g after heated at (h) 110 °C for 3 min and (i) 120 °C for 5 min, (j) PL spectra, (k) PXRD patterns, and (l) DSC thermograms of samples a−i.

Two endothermic peaks located from 124 to 160 ^o^C in the DSC thermogram of crystal TPE-4N indicate that the acetone molecules are encapsulated in crystals, which can be removed by heating. The absence of peak at 133 °C in DSC thermogram (line b in Supplementary Figure 7l) of the heated sample suggest that acetone molecules have been removed. The endothermic peak at 145 °C in the DSC thermogram of (line b in in Supplementary Figure 7l) indicates a solid-solid phase transition, thus heated single crystal was further annealed at 150 °C for 10 minutes. The PXRD pattern (line b in Supplementary Figure 7k) of the heated TPE-4N coincides well with that simulated from single crystal data of TPE-4N (line a Supplementary Figure 7k), indicating their same crystalline orders.

The DSC thermogram (line c in in Supplementary Figure 7l) and PXRD pattern (line c in Supplementary Figure 7k) of the further heated single crystal reveal that it is a new crystalline phase different from single crystal, while the new crystals are still nonemissive. Hence the residual solvent molecules in the crystals and crystalline phase do not affect the emission of crystals TPE-4N.





**Supplementary Figure 10**. (a) PL spectra of TPE-3N switching through repeating grinding and solvent fuming in five repeating cycles. Excitation wavelength: 365 nm. (b) PXRD patterns of the solvent fumed ground powders of TPE-3N before and after dried by vacuum (inset show the photos). (c) ^1^H NMR spectra of luminogen TPE-3N in DMSO-*d*_6_: ground powders after fumed by CHL and then the solvent molecules were removed by vacuum. (d) DSC curves of solvent fumed ground powders and then dried under vacuum.

The NMR spectra of the crystals were obtained from fuming ground powders, indicating the existence of solvent molecules. We tried to remove solvent molecules in the fumed ground powders under high vacuum. However, the emission of fumed ground powder does not change after removal of solvent molecules, and the unaltered crystalline essence were also suggested by the unchanged PL spectra and PXRD. Hence the residual solvent molecules in the crystals do not affect the emission of crystals TPE-3N.


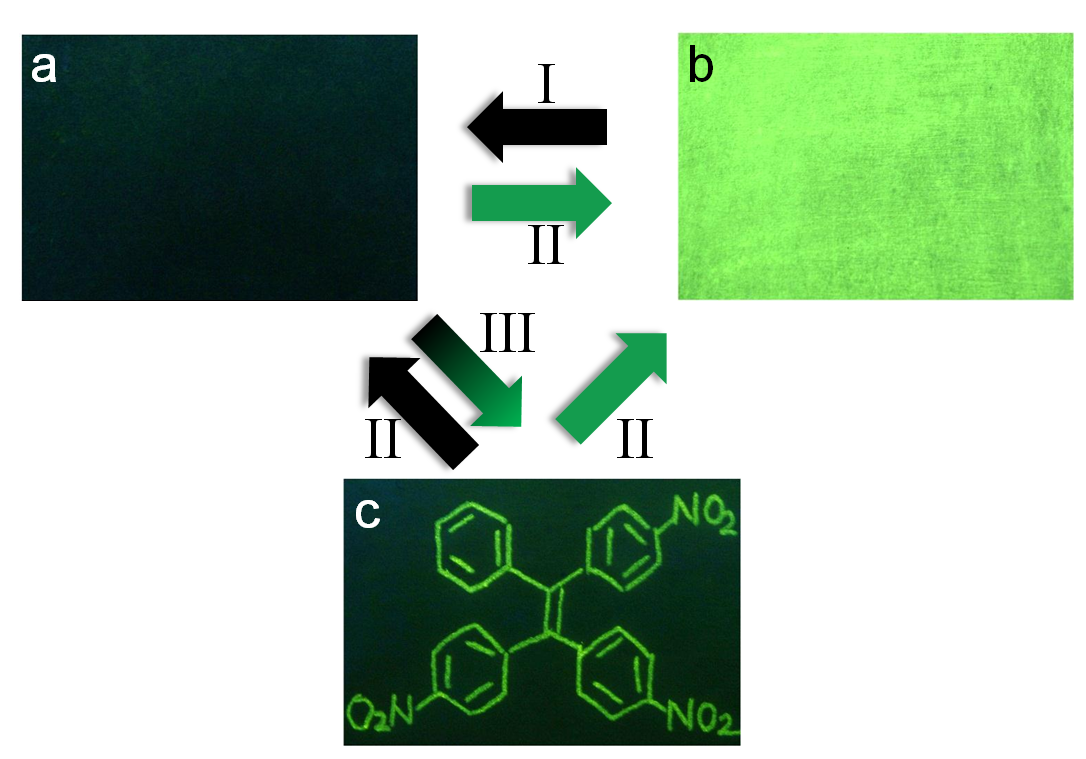


**Supplementary Figure 11.** Switching emission of TPE-3N films on a weighing paper among different luminescent states and procedures of the writing and erasing processes. Process I: fuming with DCM for 10 s; II, grinding; III, writing with a glass rod. Photos were taken on a weighing paper under UV irradiation at 365 nm.





**Supplementary Figure 12.** ^1^H NMR of luminogen TPE-4N: (a) single crystals grown from acetone solution. (b) crystals after heated at 115 °C for 1 hour. (c) ground powders fumed with acetone for 3 min and (d) then heated at 110 °C for 3 min.

The ^1^H-NMR spectra of the crystals recrystallized from acetone solution and ground powders fumed by acetone indicate the existence of solvent molecules. After heated, acetone molecules in crystals were removed, which is indicated by disappearing of the resonance peak at 2.09 ppm in ^1^H NMR.


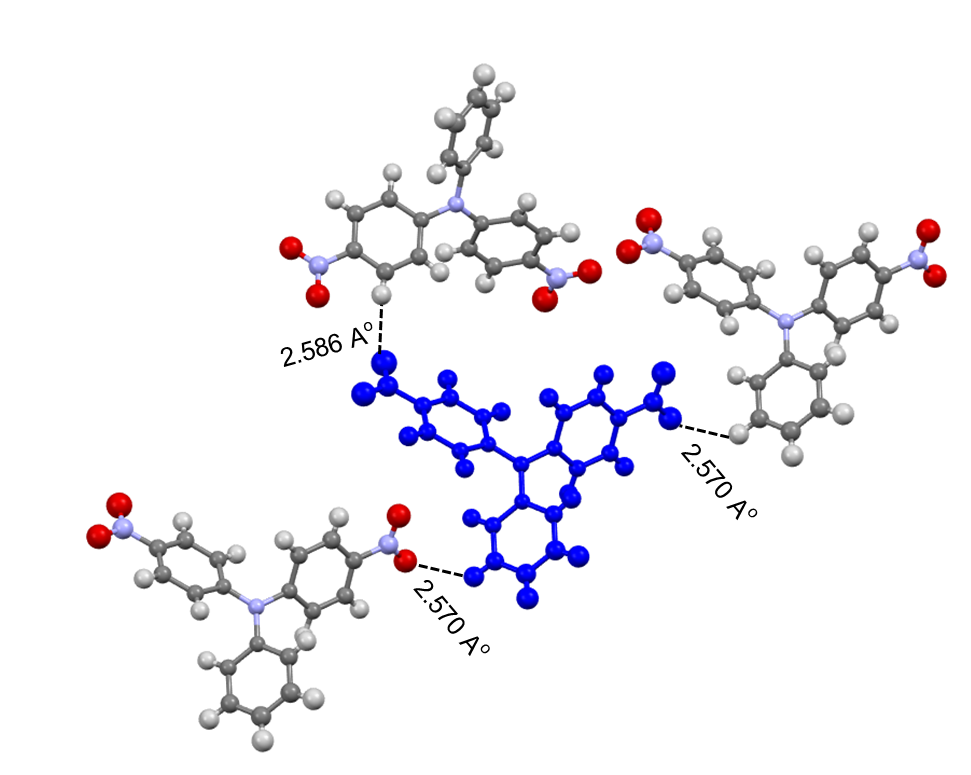


**Supplementary Figure 13**. Intermolecular interactions in TPA-2N single crystal.


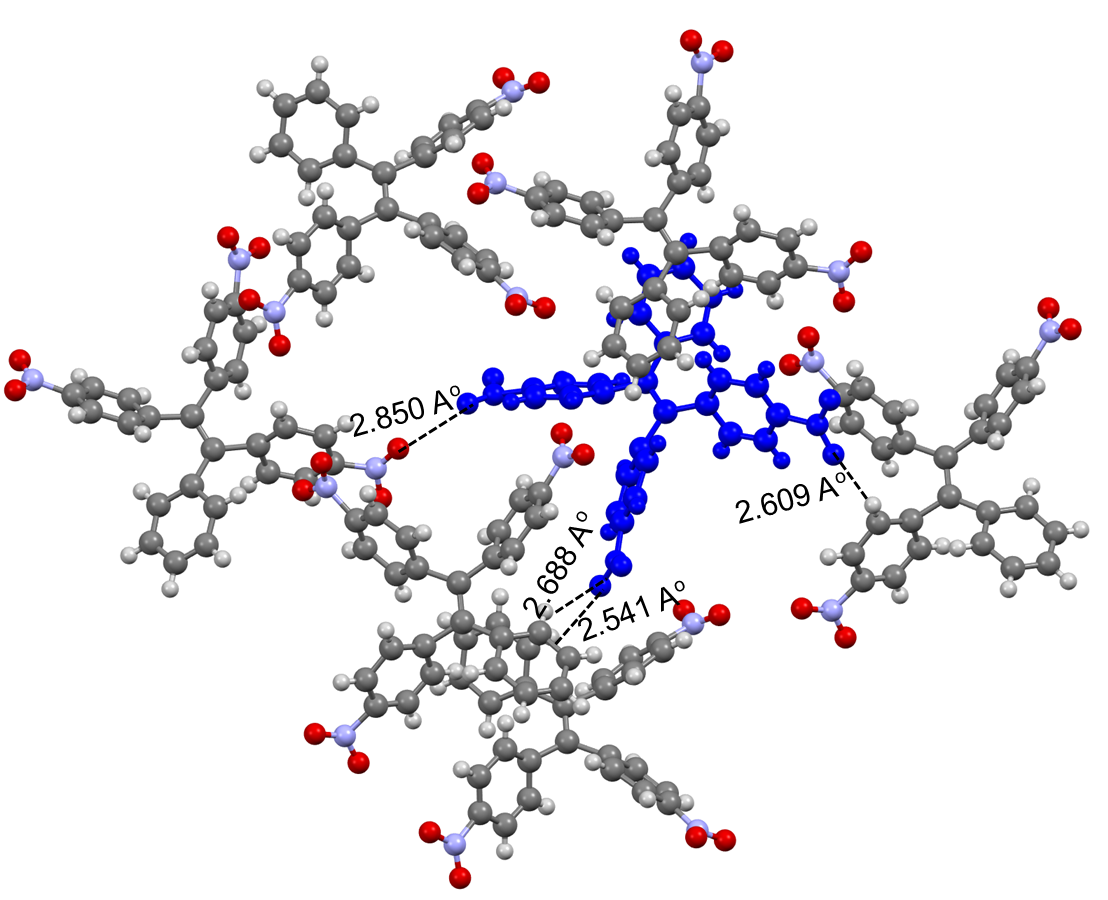


**Supplementary Figure 14**. Intermolecular interactions in TPE-3N single crystal.


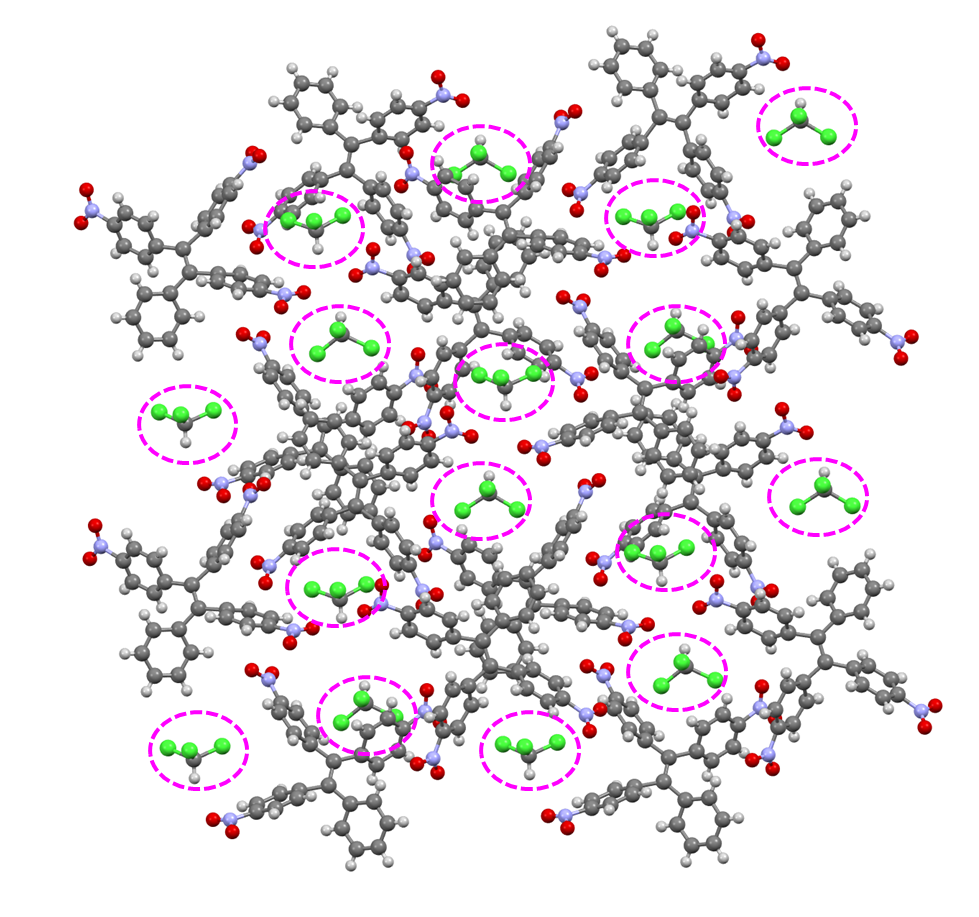


**Supplementary Figure 15**. Chloroform molecules in TPE-3N crystal (marked by pink cycle).


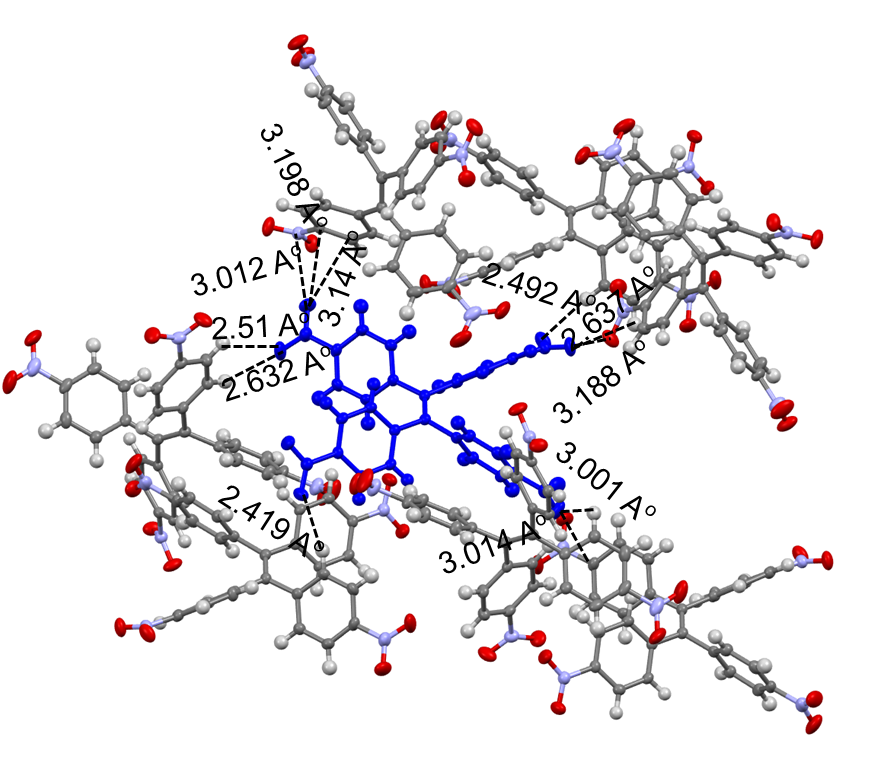


**Supplementary Figure 16**. Intermolecular interactions in TPE-4N single crystal.





**Supplementary Figure 17**. (a) UV-vis absorption spectra of spin-coating film of TPA-1N, (b) crystalline and ground powders of TPA-1N performed by diffuse reflectance mode.

The computational models were built from the crystal structures shown below. The quantum mechanics/molecular mechanics (QM/MM) method was implemented to deal with the electronic structures in crystal by virtue of ChemShell 3.5.^2^, interfacing Turbomole 6.5^3^ for QM and DL_POLY^4^ with the general Amber force field (GAFF)^5^ for MM. The atomic partial charges were generated by the restrained electrostatic potential (RESP)^6^ method. Molecular geometry optimizations were performed for the ground state (S_0_) at the level of B3LYP/6-31G*. The excitation energies of singlet and triplet states were calculated using TDDFT method. At the same level, the spin-orbit coupling between singlet and triplet states were given by Beijing Density Function (BDF) program.^7-9^


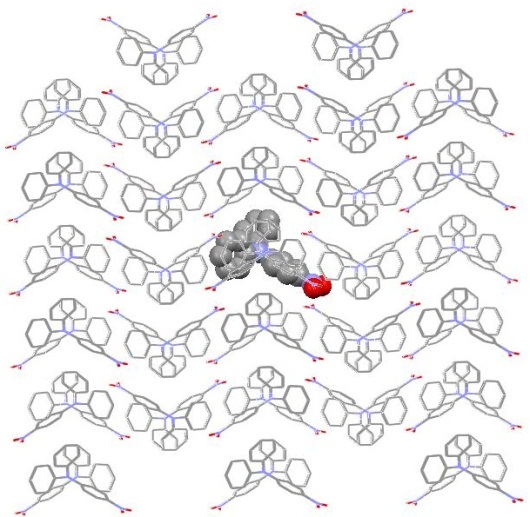


**Supplementary Figure 18** QM/MM model taking TPA-1N as an example: one central QM molecule for the higher layer and the surrounding 124 MM molecules for the lower layer.


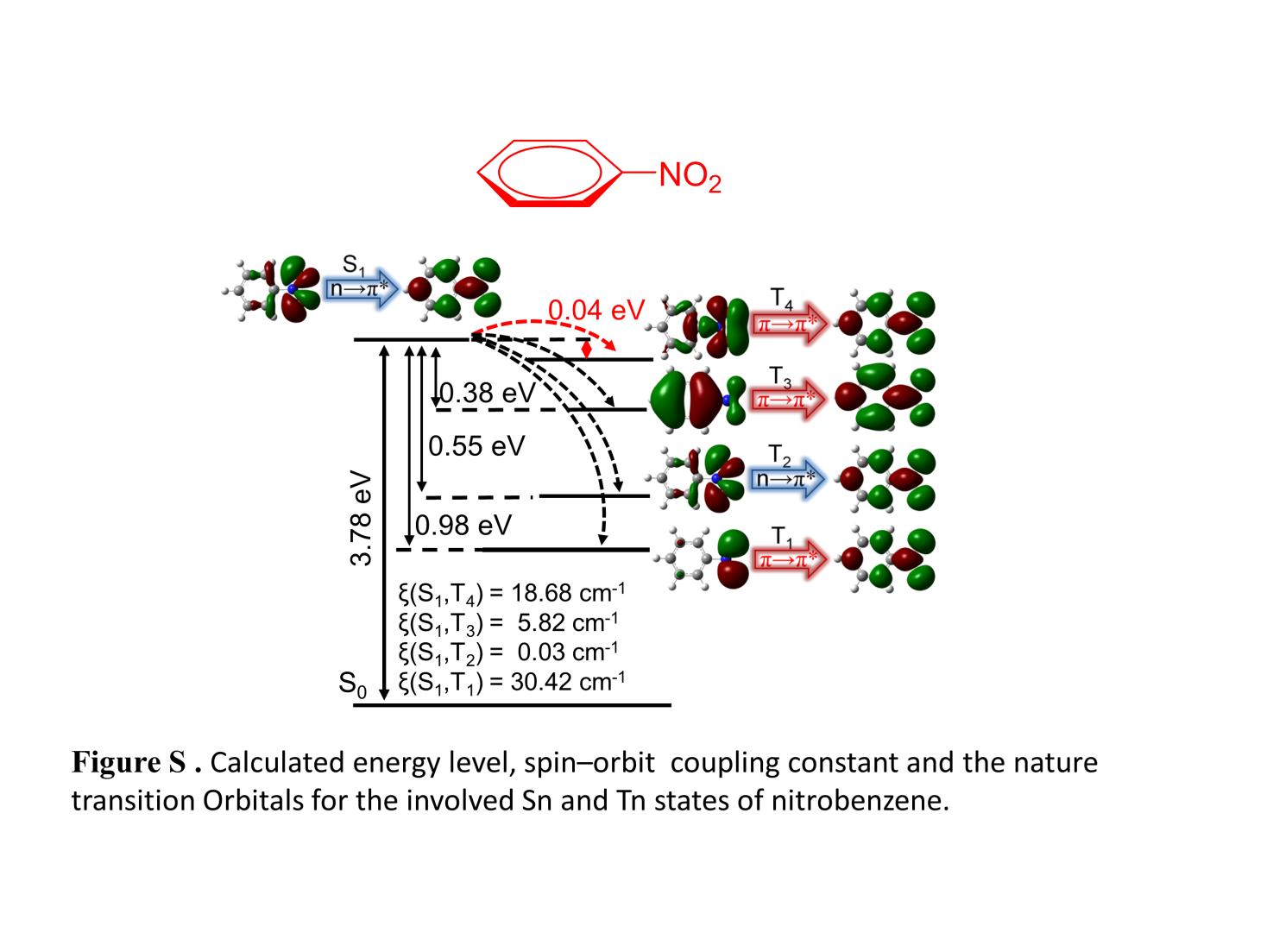


**Supplementary Figure 19.** Calculated energy levels, spin-orbit coupling constants and the nature transition orbitals for the involved S_1_ and T_n_ states of nitrobenzene at B3LYP-6-31G* level.


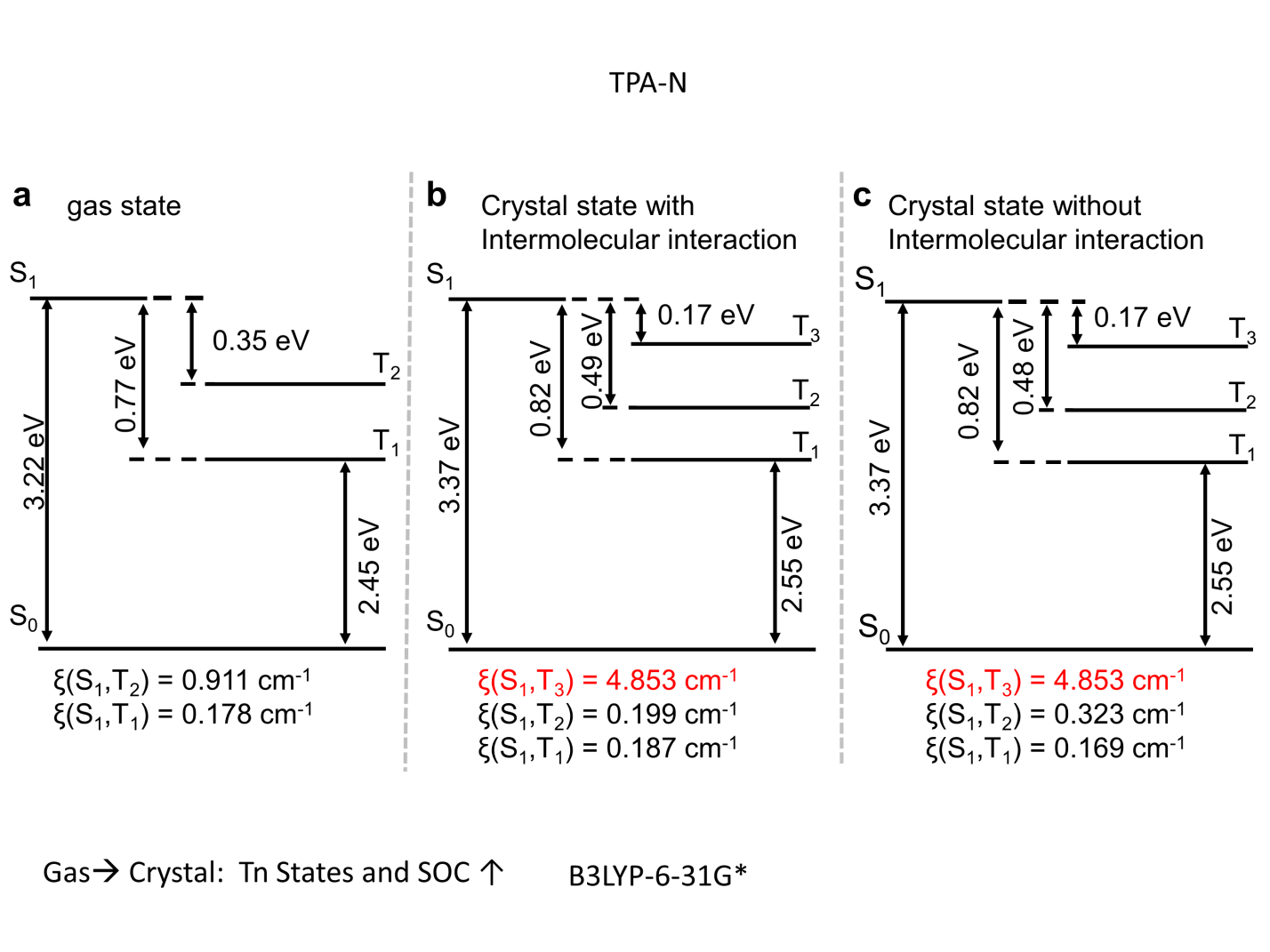


**d**  **e**


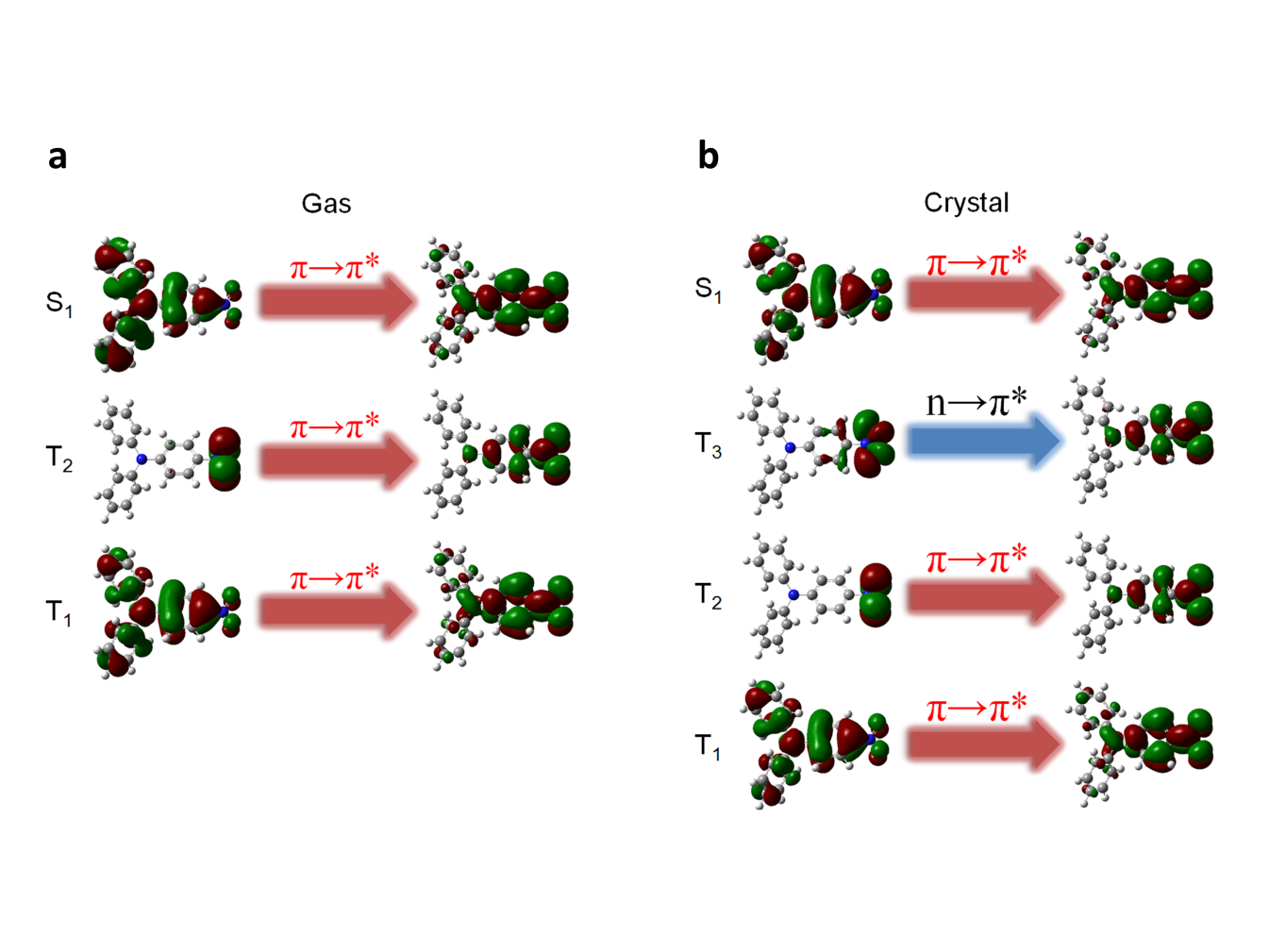


**Supplementary Figure 20**. Calculated energy levels and spin-orbit coupling constants for the involved S_1_ and T_n_ states of TPA-1N in gas state (a), crystal state with intermolecular interaction (b) based on QM/MM method and crystal state without intermolecular interaction (c) at B3LYP-6-31G* level. Nature transition orbitals for the involved S_1_ and T_n_ states of TPA-1N in gas state (d) and crystal state (e) at B3LYP-6-31G* level.


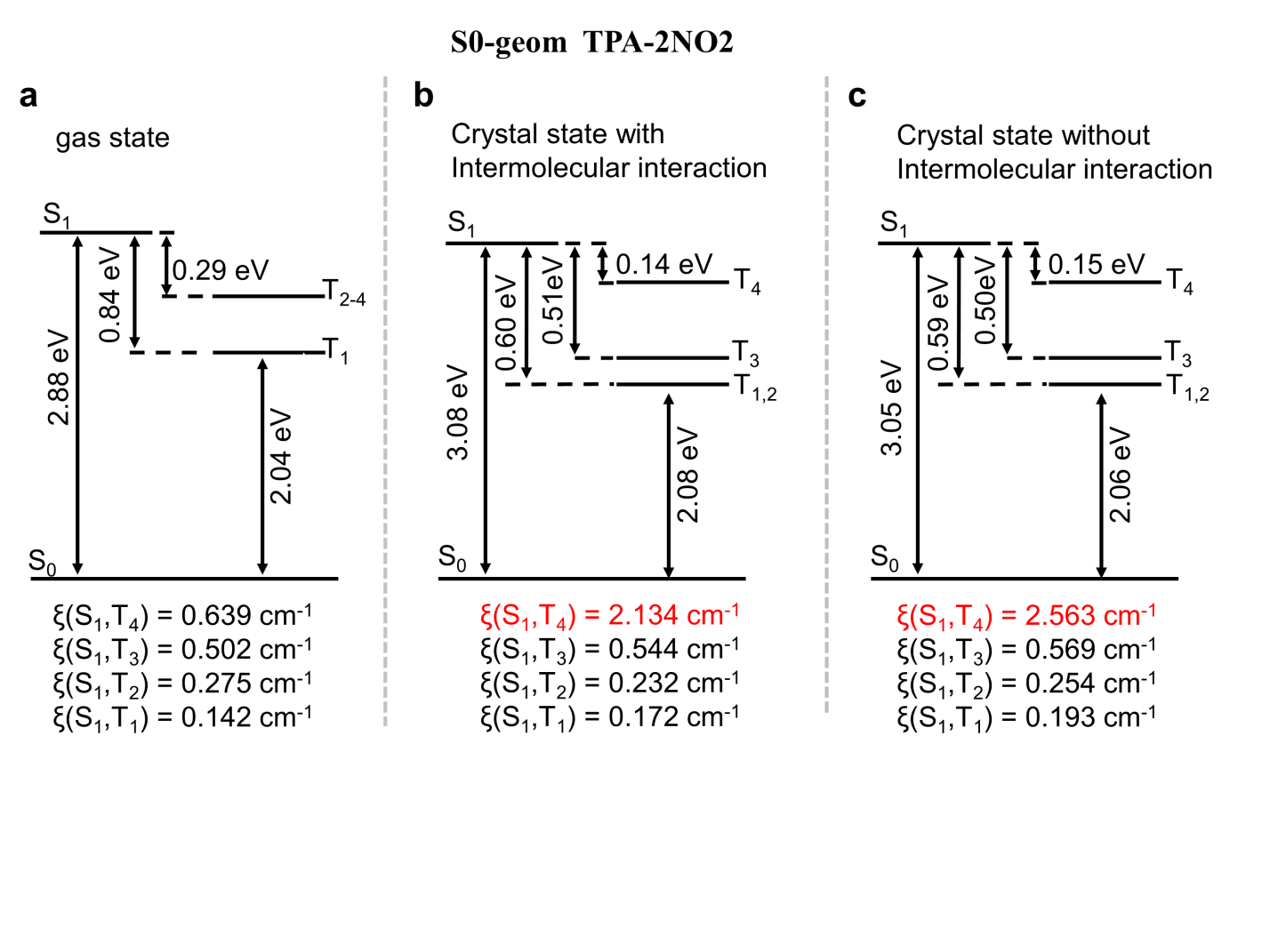


**Supplementary Figure 21**. Calculated energy levels and spin-orbit coupling constants for the involved S_1_ and T_n_ states of TPA-2N in gas state (a), crystal state with intermolecular interaction (b) based on QM/MM method and crystal state without intermolecular interaction (c) at B3LYP-6-31G* level.


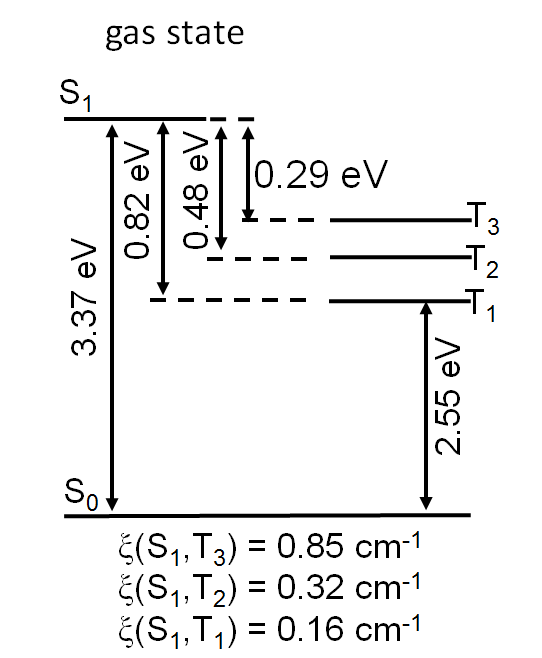


**Supplementary Figure 22**. Calculated energy levels and spin-orbit coupling constants for the involved S_1_ and T_n_ states of TPE-1N in gas state at B3LYP-6-31G* level.


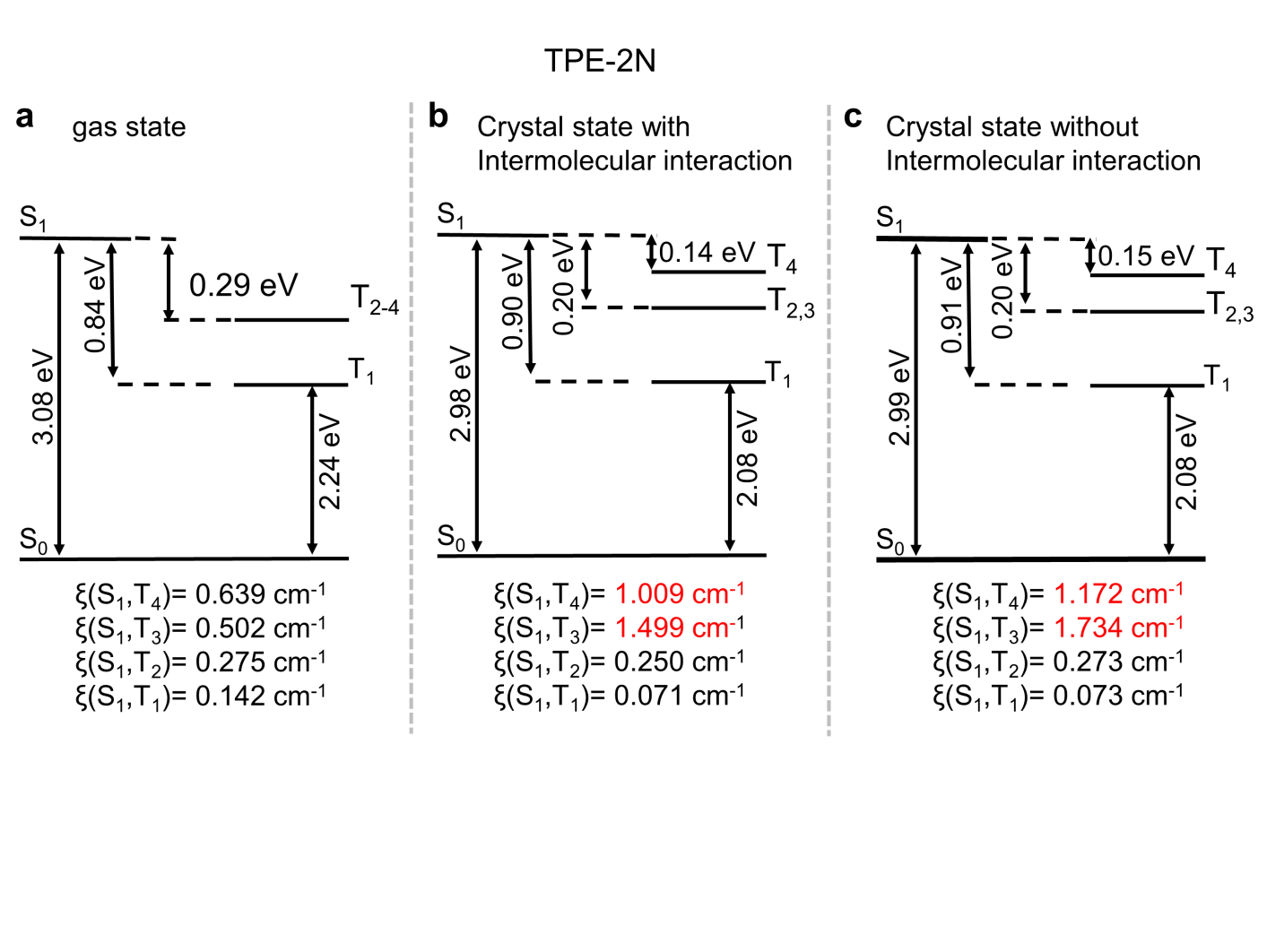


**Supplementary Figure 23**. Calculated energy levels and spin-orbit coupling constants for the involved S_1_ and T_n_ states of TPE-2N in gas state (a), crystal state with intermolecular interaction (b) based on QM/MM method and crystal state without intermolecular interaction (c) at B3LYP-6-31G* level.


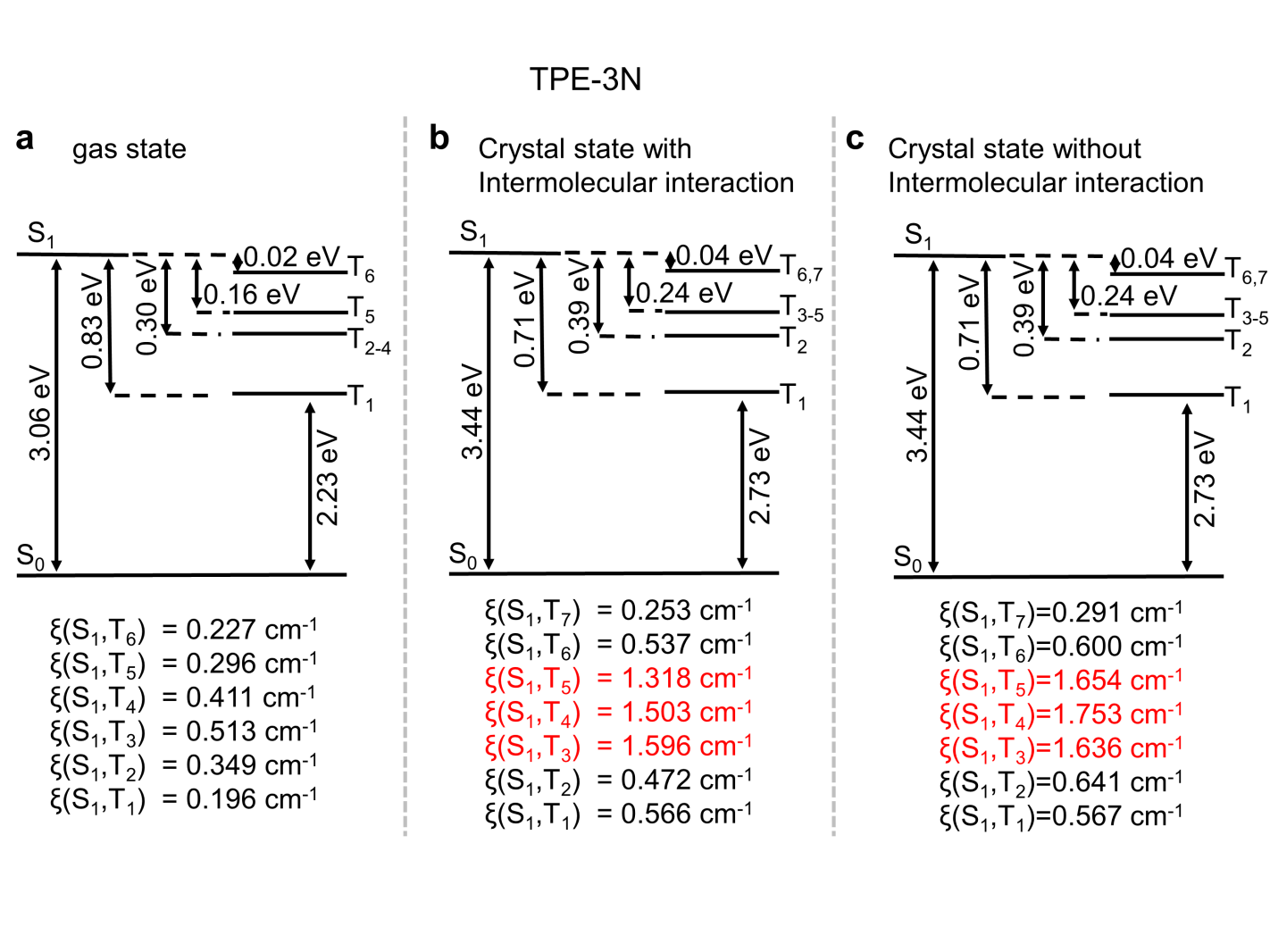


**Supplementary Figure 24**. Calculated energy levels and spin-orbit coupling constants for the involved S_1_ and T_n_ states of TPE-3N in gas state (a), crystal state with intermolecular interaction (b) based on QM/MM method and crystal state without intermolecular interaction (c) at B3LYP-6-31G* level.


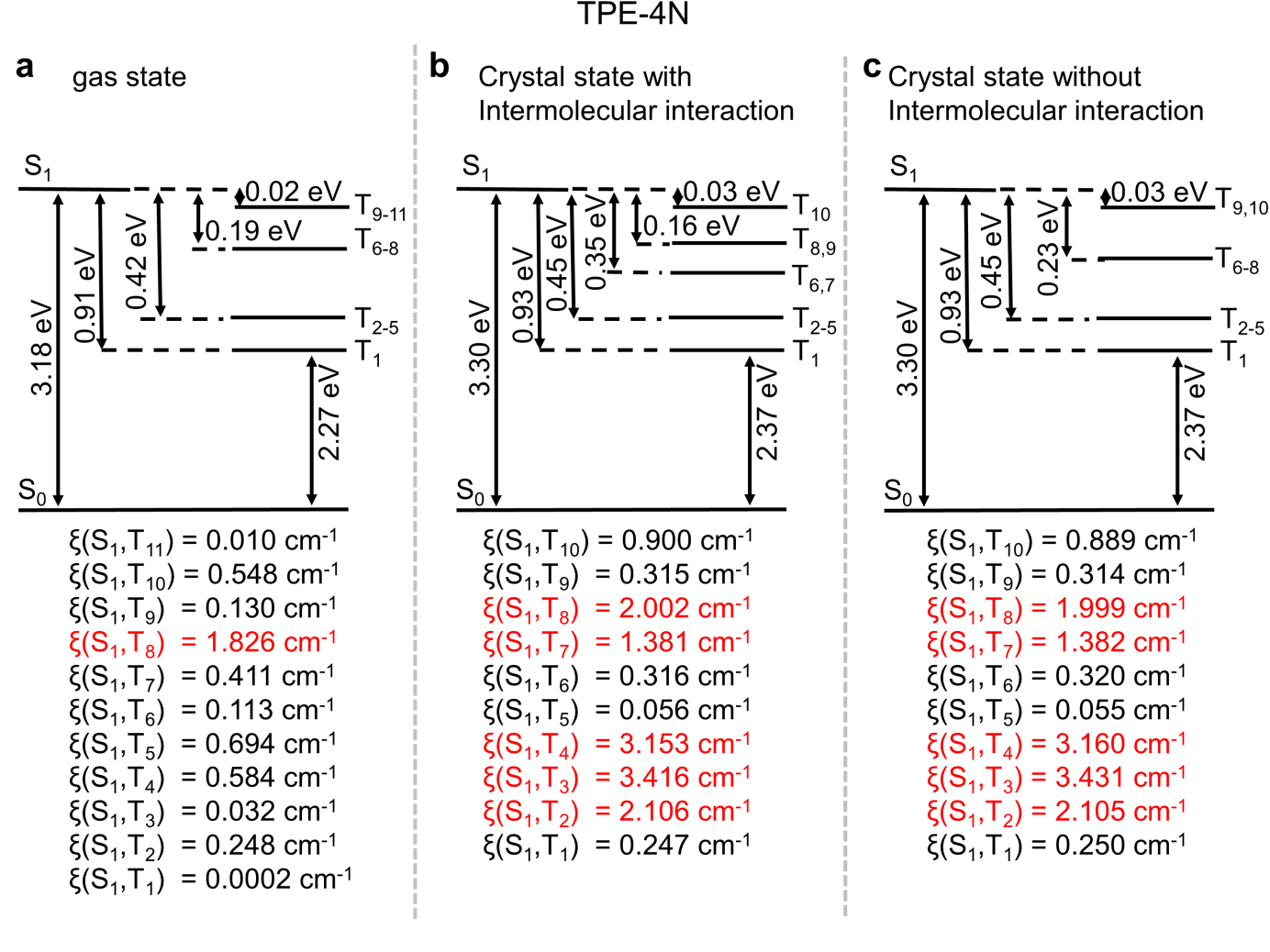


**Supplementary Figure 25**. Calculated energy levels and spin-orbit coupling constants for the involved S_1_ and T_n_ states of TPE-4N in gas state (a), crystal state with intermolecular interaction (b) based on QM/MM method and crystal state without intermolecular interaction (c) at B3LYP-6-31G* level.


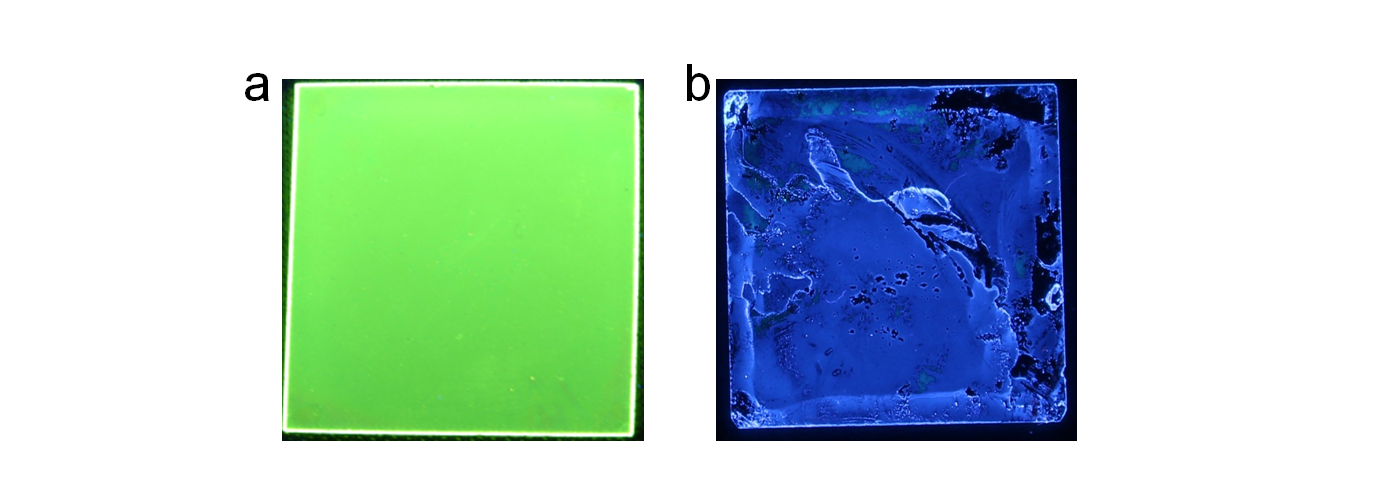


**Supplementary Figure 26**. Spin-coating films of TPE-4N (a) and TPE (b) with the same fabrication process.


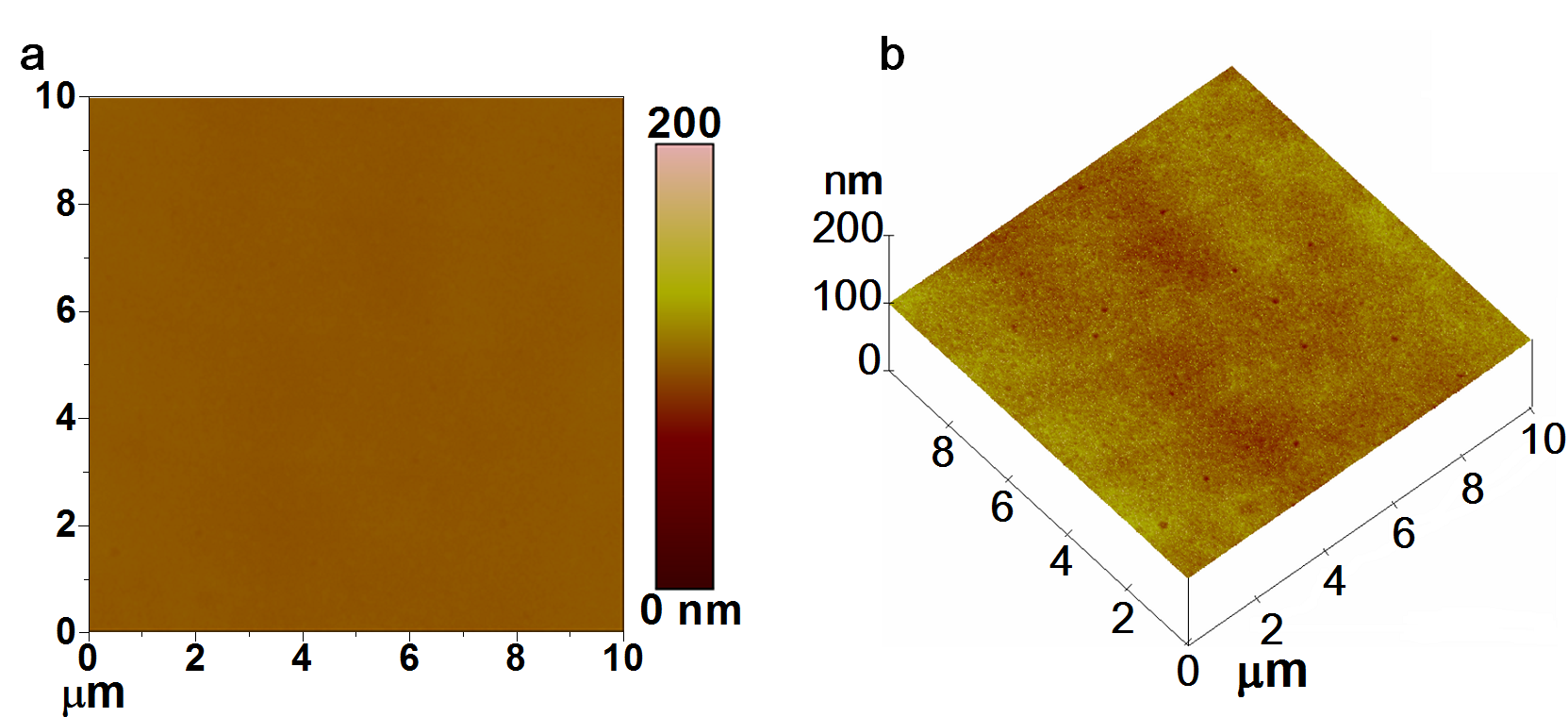


**Supplementary Figure 27**. AFM images of freshly prepared film of TPE-4N.

**
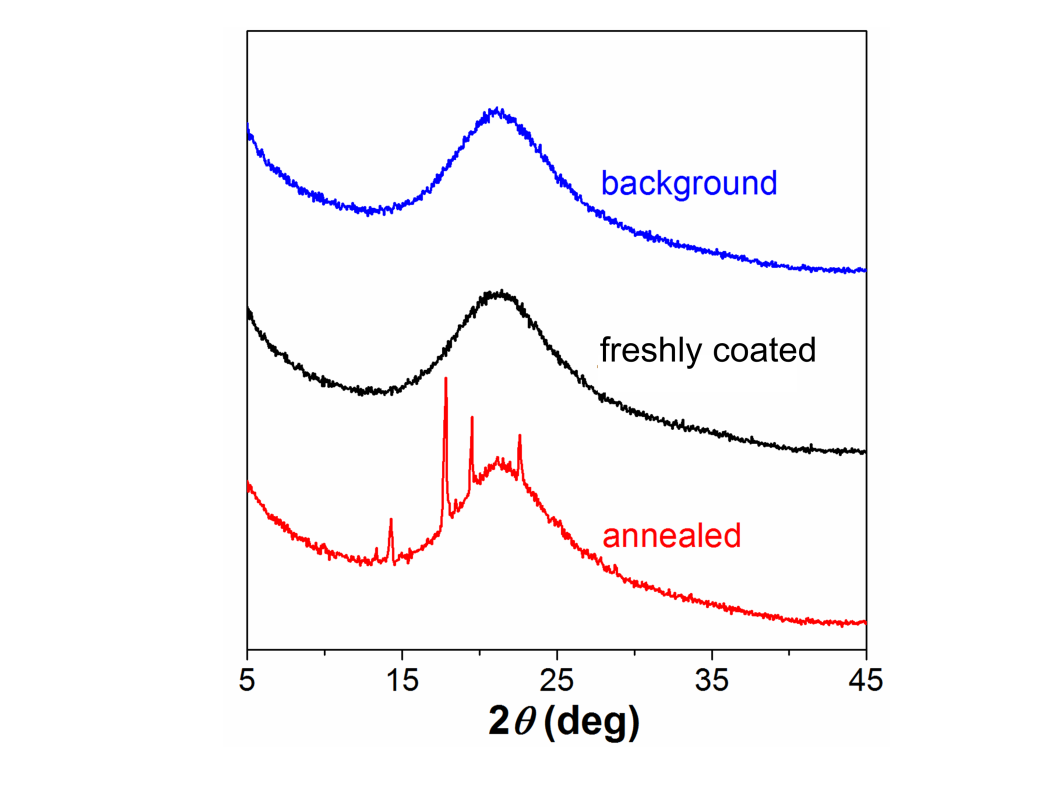
**

**Supplementary Figure 28**. PXRD patterns of freshly coated and annealed films of TPE-4N.


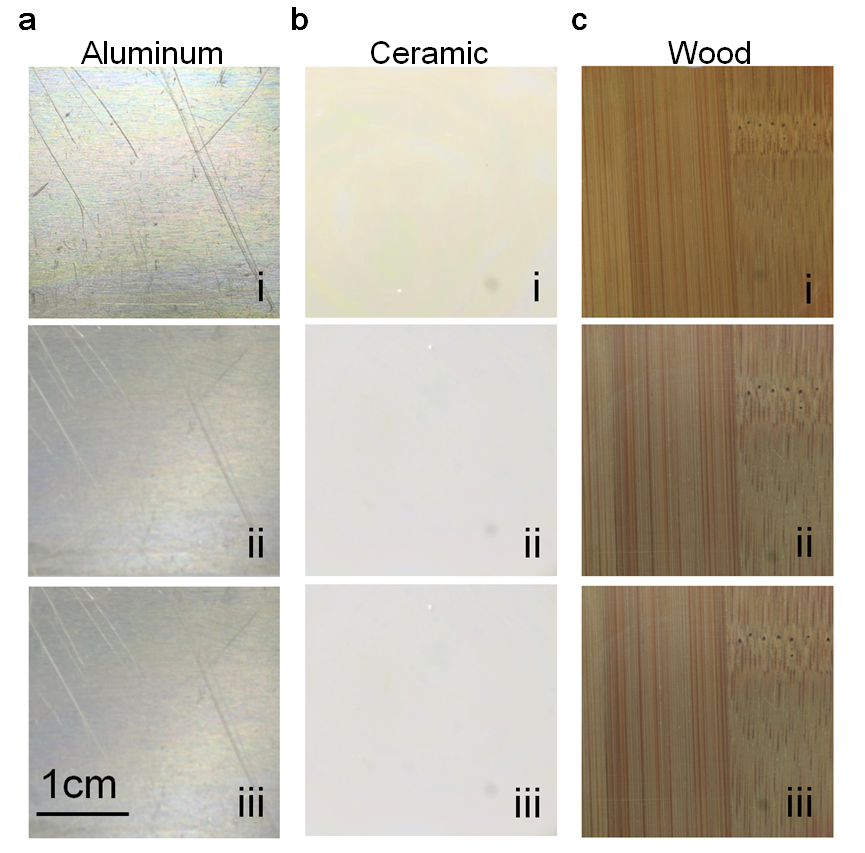


**Supplementary Figure 29**. Room light photos of aluminum (a), ceramic (b) and wooden (c) substrates coated with TPE-4N: i, freshly brush coating film; ii, annealed film; iii, annealed film pressed with finger.


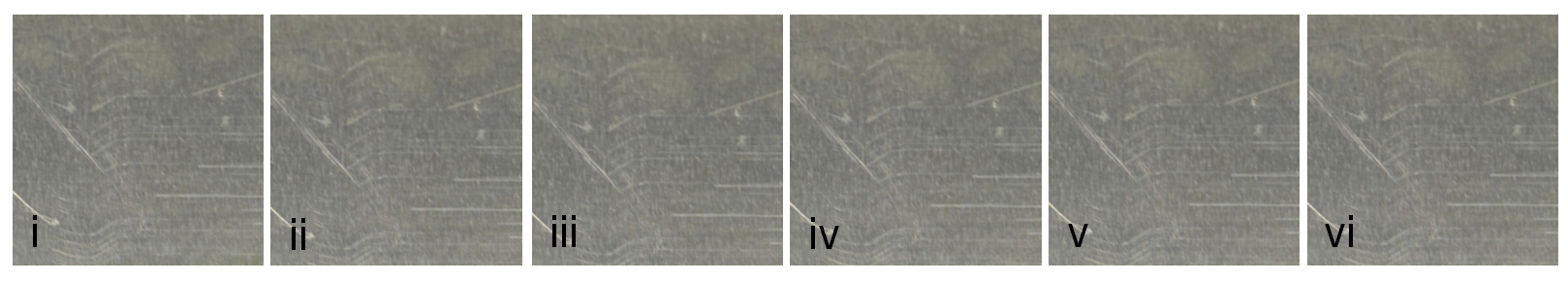


**
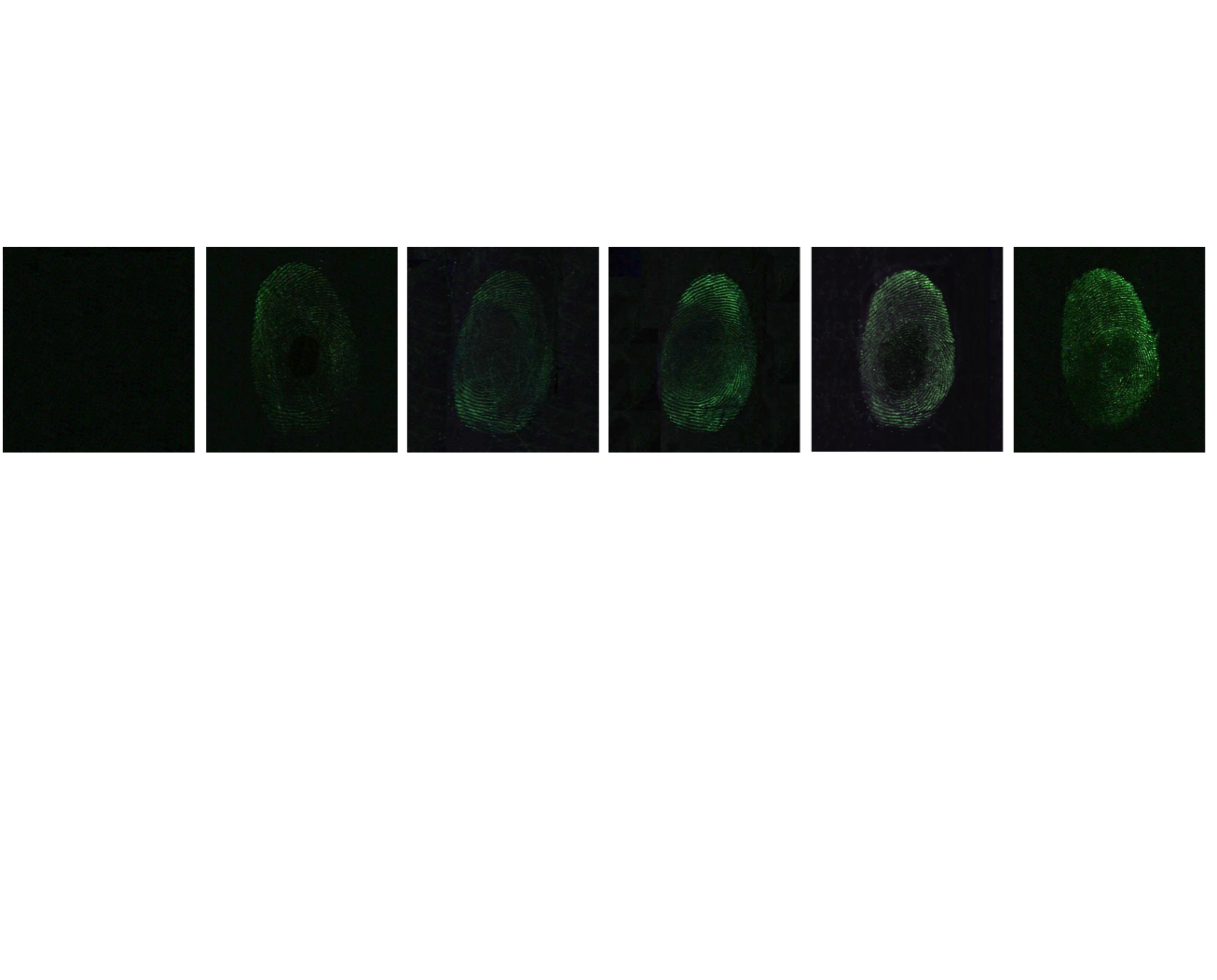
**

**Supplementary Figure 30**. (Up) Room light and (down) fluorescent photos of aluminum coated with TPE-4N with increased finger pressure from 0 to 0.98 MPa.


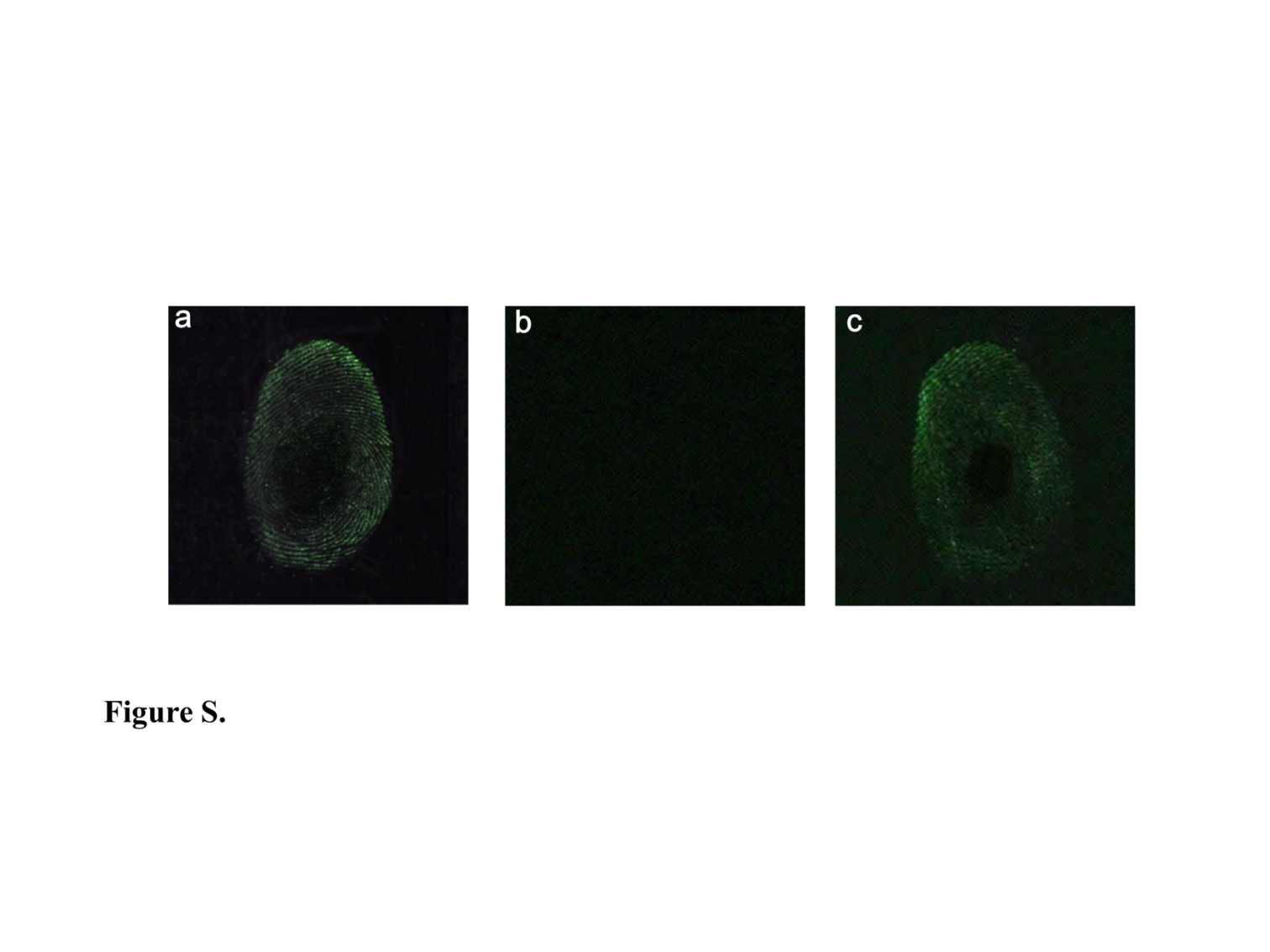


**Supplementary Figure 31**. Luminescent photos of fingerprint on aluminum: (a) pressed film, (b) recovered film (c) re-pressed film.





**Supplementary Figure 32.** (a) Chemical structure of TPE-BP. (b) PL spectra of TPE-BP in solution (acetonitrile, *f*_w_ = 0) and aggregate (acetonitrile / water mixtures, *f*_w_ = 90). Inset: fluorescent photos of acetonitrile/water mixture with *f*_w_ = 0 and 90. Luminogen concentration: 1×10^−5^ M. (c) Crystalline (left) and amorphous (right) photos of TPE-BP under room and UV light (Luminescence quantum yields and contrast ratios are shown under the photos). (d) PL spectra and fluorescent photos of ground and fumed TPE-BP powders.


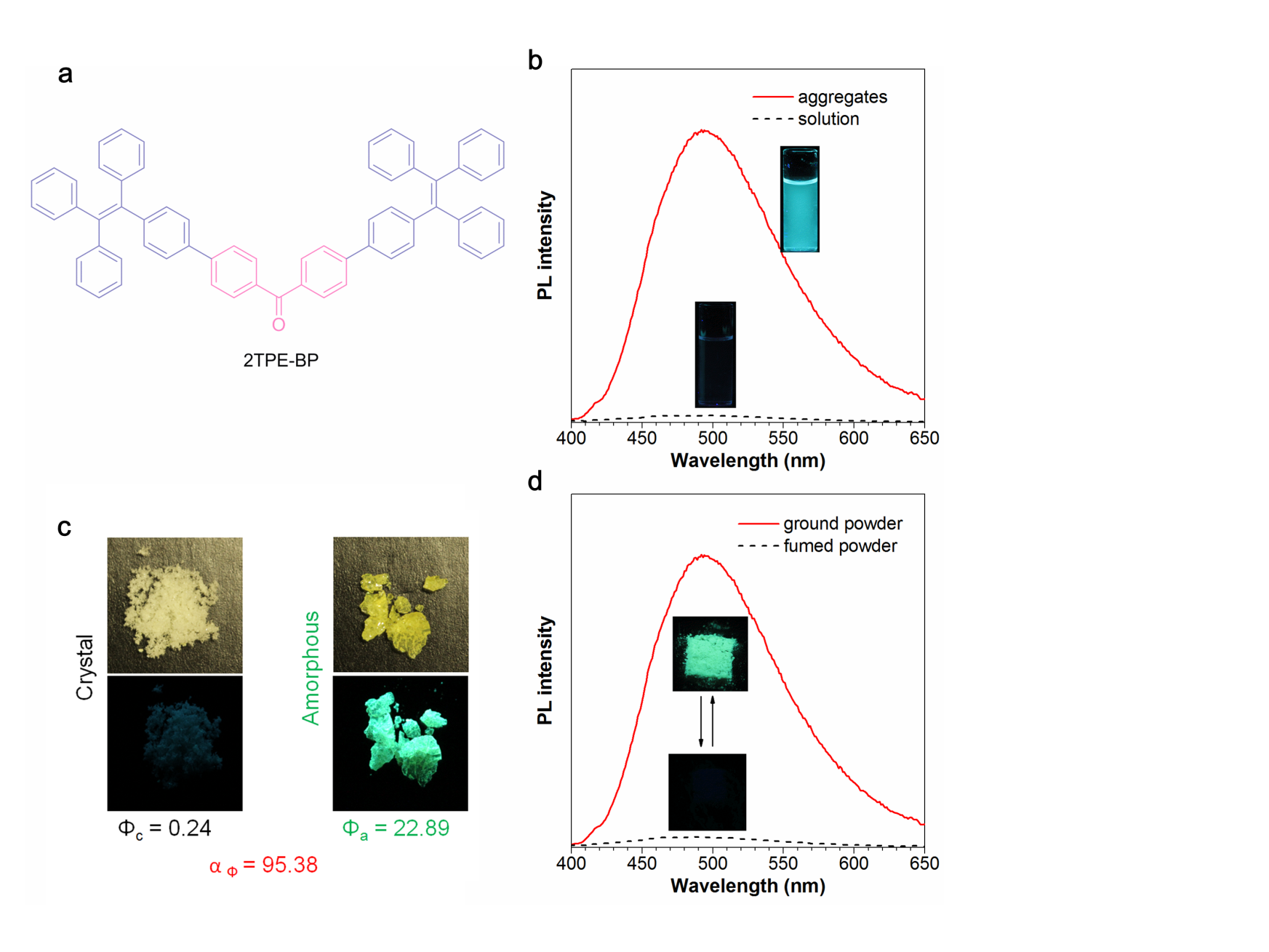


**Supplementary Figure 33.** (a) Chemical structure of 2TPE-BP. (b) PL spectra of 2TPE-BP in solution (acetonitrile, *f*_w_ = 0) and aggregate (acetonitrile / water mixtures, *f*_w_ = 90). Inset: fluorescent photos of acetonitrile/water mixture with *f*_w_ = 0 and 90. Luminogen concentration: 1×10^−5^M. (c) Crystalline (left) and amorphous (right) photos of 2TPE-BP under room and UV light (Luminescence quantum yields and contrast ratios are shown under the photos). (d) PL spectra and fluorescent photos of ground and fumed 2TPE-BP powders.

**Supplementary Tables**

**Supplementary Table 1.** Emission properties of samples in aggregates and film states.


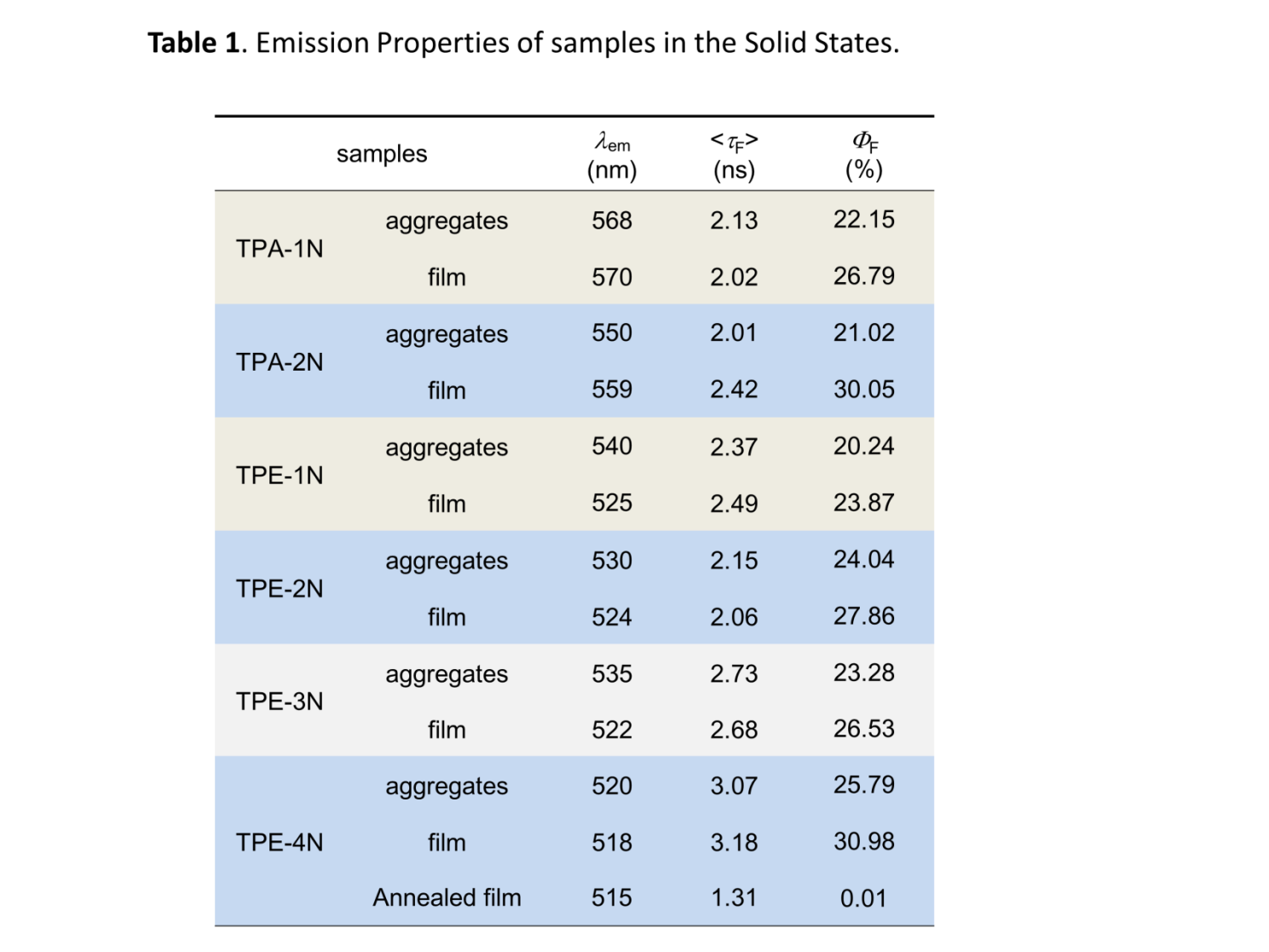


**Supplementary Table 2.** Emission properties of samples in crystalline and amorphous states.


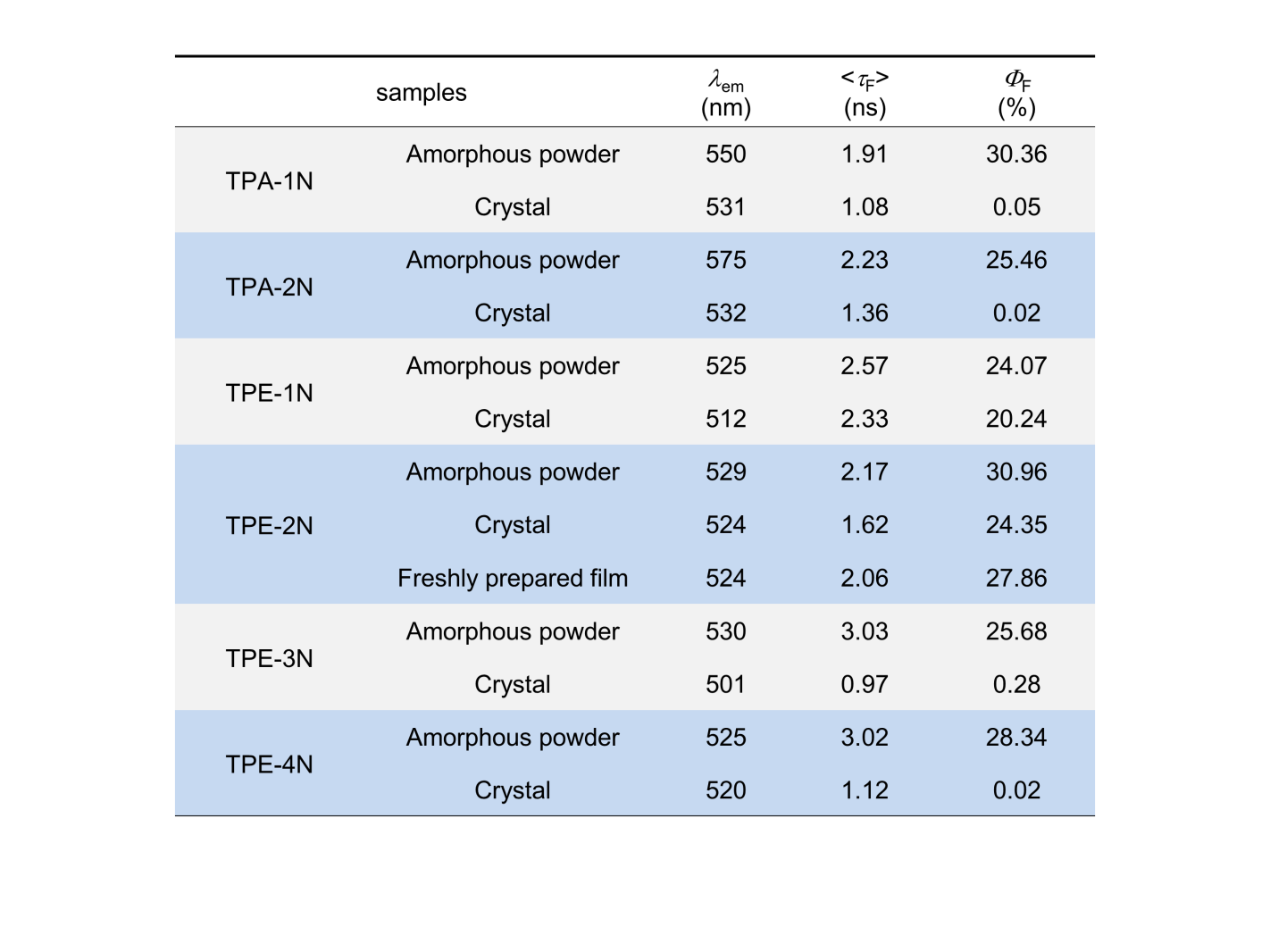


**Supplementary Table 3.** Crystal Data and Structure Refinement for TPE-4N.

| Empirical formula | C26 H16 N4 O8 |
| --- | --- |
| Formula weight | 512.43 |
| Temperature | 110(2) K |
| Wavelength | 0.71073 Å |
| Crystal system | Tetragonal |
| Space group | P4(2)/n |
| Unit cell dimensions | a = 19.7077(10) Å alpha = 90 deg.  b = 19.7077(10) Å beta = 90 deg.  c = 13.3736(15) Å gamma = 90 deg. |
| Volume | 5194.2(7) Å^3^ |
| Z | 8 |
| Density (calculated) | 1.311 Mg/m^3^ |
| Absorption coefficient | 0.100 mm^-1^ |
| F(000)  Crystal size | 2112  0.40 x 0.35 x 0.31 mm^3^ |
| Theta range for data collection | 2.07 to 25.24 |
| Index ranges | -16<=h<=16, 0<=k<=23, 0<=l<=16 |
| Reflections collected | 4704 |
| Independent reflections | 4704 [R(int) = 0.0000] |
| Absorption correction | Semi-empirical from equivalents |
| Max. and min. transmission | 0.9740 and 0.9687 |
| Refinement method | Full-matrix least-squares on F2 |
| Data / restraints / parameters | 4704 / 0 / 343 |
| Goodness-of-fit on F2 | 1.099 |
| Final R indices [I>2sigma(I)] | R1 = 0.0423, wR2 = 0.1179 |
| R indices (all data) | R1 = 0.0544, wR2 = 0.1243 |
| Largest diff. peak and hole | 0.422 and -0.192 e.Å^-3^ |

**Supplementary Table 4.** Crystal Data and Structure Refinement for TPE-3N.

| Empirical formula | C_26_ H_17_ N_3_ O_6_ |
| --- | --- |
| Formula weight | 586.79 |
| Temperature | 110(2) K |
| Wavelength | 0.71073 Å |
| Crystal system | Orthorhombic |
| Space group | Fdd2 |
| Unit cell dimensions | a = 34.447(4) Å alpha = 90 deg.  b = 37.636(5) Å beta = 90 deg.  c = 8.8988(11) Å gamma = 90 deg. |
| Volume | 11537(2) Å^3^ |
| Z | 16 |
| Density (calculated) | 1.351 Mg/m^3^ |
| Absorption coefficient | 0.362 mm^-1^ |
| F(000)  Crystal size | 4800  0.46 x 0.40 x 0.24 mm^3^ |
| Theta range for data collection | 2.16 to 25.24 |
| Index ranges | 0<=h<=41, 0<=k<=44, -10<=l<=9 |
| Reflections collected | 4950 |
| Independent reflections | 4950 [R(int) = 0.0000] |
| Absorption correction | Semi-empirical from equivalents |
| Max. and min. transmission | 0.9182 and 0.8512 |
| Refinement method | Full-matrix least-squares on F2 |
| Data / restraints / parameters | 4950 / 285 / 379 |
| Goodness-of-fit on F2 | 1.078 |
| Final R indices [I>2sigma(I)] | R1 = 0.0999, wR2 = 0.2746 |
| R indices (all data) | R1 = 0.1184, wR2 = 0.2865 |
| Largest diff. peak and hole | 0.388 and -0.534e.Å^-3^ |

**Supplementary Table 5.** Crystal Data and Structure Refinement for TPE-2N.

| Empirical formula | C26 H18 N2 O4 |
| --- | --- |
| Formula weight | 422.42 |
| Temperature | 110(2) K |
| Wavelength | 0.71073 Å |
| Crystal system | Monoclinic |
| Space group | P2(1)/c |
| Unit cell dimensions | a = 10.4723(16) Å alpha = 90 deg.  b = 8.6413(14) Å beta = 90 deg.  c = 22.938(4) Å gamma = 90 deg. |
| Volume | 2065.2(6) (7) Å^3^ |
| Z | 4 |
| Density (calculated) | 1. 337 Mg/m^3^ |
| Absorption coefficient | 0.089 mm^-1^ |
| F(000)  Crystal size | 880  0.45 x 0.33 x 0.21 mm^3^ |
| Theta range for data collection | 1.95 to 27.58 |
| Index ranges | -10<=h<=13, -11<=k<=8, -29<=l<=29 |
| Reflections collected | 11641 |
| Independent reflections | 4699 [R(int) = 0.0383] |
| Absorption correction | Semi-empirical from equivalents |
| Max. and min. transmission | 0.9808 and 0.9594 |
| Refinement method | Full-matrix least-squares on F2 |
| Data / restraints / parameters | 4699 / 0 / 289 |
| Goodness-of-fit on F2 | 1.033 |
| Final R indices [I>2sigma(I)] | R1 = 0.0454, wR2 = 0.0934 |
| R indices (all data) | R1 = 0.0695, wR2 = 0.1033 |
| Largest diff. peak and hole | 0.255 and -0.221 e.Å^-3^ |

**Supplementary Table 6.** Crystal Data and Structure Refinement for TPA-1N.

| Empirical formula | C18 H14 N2 O2 |
| --- | --- |
| Formula weight | 290.31 |
| Temperature | 173(2) K |
| Wavelength | 0.71073 Å |
| Crystal system | orthorhombic |
| Space group | Pbca |
| Unit cell dimensions | a = 12.9061(6) Å alpha = 90 deg.  b = 12.9356(8) Å beta = 90 deg.  c = 17.2834(9) Å gamma = 90 deg. |
| Volume | 2885.4(3) Å^3^ |
| Z | 8 |
| Density (calculated) | 1. 359 Mg/m^3^ |
| Absorption coefficient | 0.093 mm^-1^ |
| F(000)  Crystal size | 1216  0.40 x 0.30 x 0.20 mm^3^ |
| Theta range for data collection | 1.95 to 27.58 |
| Index ranges | -17<=h<=10, -17<=k<=10, -22<=l<=20 |
| Reflections collected | 11059 |
| Independent reflections | 4699 [R(int) = 0.0383] |
| Absorption correction | Semi-empirical from equivalents |
| Max. and min. transmission | 0.9808 and 0.9594 |
| Refinement method | Full-matrix least-squares on F2 |
| Data / restraints / parameters | 3342 / 0 /199 |
| Goodness-of-fit on F2 | 1.017 |
| Final R indices [I>2sigma(I)] | R1 = 0.0454, wR2 = 0.0937 |
| R indices (all data) | R1 = 0.0695, wR2 = 0.1084 |
| Largest diff. peak and hole | 0.157 and -0.221 e.Å^-3^ |

**Supplementary References**

1 Zhao, Z. *et al*. Creation of highly efficient solid emitter by decorating pyrene core with AIE-active tetraphenylethene peripheries. *Chem. Commun.* **46**, 2221-2223 (2010).

2 Sherwood, P. *et al*. QUASI: A General Purpose Implementation of the QM/MM Approach and Its Application to Problems in Catalysis. *J. Mol. Struct.: Theochem*. **632**, 1-28 (2003).

3 Ahlrichs, R.; Bär, M.; Häser, M.; Horn, H.; Kölmel, C. Electronic Structure Calculations on Workstation Computers: The Program System Turbomole. *Chem. Phys. Lett.* **162**, 165-169 (1989).

4 Smith, W.; Forester, T. R. DL_POLY_2.0: A General-Purpose Parallel Molecular Dynamics Simulation Package. *J. Mol. Graph.* **14**, 136-141 (1996).

5 Wang, J.; Wolf, R. M.; Caldwell, J. W.; Kollman, P. A.; Case, D. A. Development and Testing of a General Amber Force Field. *J. Comput. Chem.* **25**, 1157-1174 (2004).

6 Bayly, C. I.; Cieplak, P.; Cornell, W.; Kollman, P. A. A Well-Behaved Electrostatic Potential Based Method Using Charge Restraints for Deriving Atomic Charges: The RESP Model. *J. Phys. Chem.* **97**, 10269-10280 (1993).

7 Liu, W.; Wang, F.; Dai, D.; Li, L.; Dolg, M. The Beijing Four-Component Density Functional Program Package (BDF) And Its Application to EuO, EuS, YbO and YbS. *Theor. Chem. Acc.* **96**, 75-83 (1997).

8 Liu, W.; Hong, G.; Li, L. The Beijing Density Functional (BDF) Program Package: Methodologies and Applications. *J. Theor. Comput. Chem.* **2**, 257-272 (2003).

9 Hirao, K.; Ishikawa, Y. Recent Advances in Computational Chemistry. *World Scientific, Singapore*. **5**, 257 (2004).

10 Fateminia, S. M. *et al*. Nanocrystallization: A Unique Approach to Yield Bright Organic Nanocrystals for Biological Applications. *Adv. Mater.* **29**, 1604100 (2017).
